# Safety of SARS-CoV-2 vaccines: a systematic review and meta-analysis of randomized controlled trials

**DOI:** 10.1186/s40249-021-00878-5

**Published:** 2021-07-05

**Authors:** Musha Chen, Yue Yuan, Yiguo Zhou, Zhaomin Deng, Jin Zhao, Fengling Feng, Huachun Zou, Caijun Sun

**Affiliations:** 1 School of Public Health (Shenzhen), Sun Yat-Sen University, Shenzhen, China; 2 Key Laboratory of Tropical Disease Control (Sun Yat-Sen University), Ministry of Education, Guangzhou, China

**Keywords:** COVID-19 vaccine, Safety, Adverse events following immunization, Randomized controlled trial, Meta-analysis

## Abstract

**Background:**

Various modalities of vaccines against coronavirus disease 2019 (COVID-19), based on different platforms and immunization procedures, have been successively approved for marketing worldwide. A comprehensive review for clinical trials assessing the safety of COVID-19 vaccines is urgently needed to make an accurate judgment for mass vaccination.

**Main text:**

A systematic review and meta-analysis was conducted to determine the safety of COVID-19 vaccine candidates in randomized controlled trials (RCTs). Data search was performed in PubMed, Embase, Cochrane library, Scopus, Web of Science, and MedRxiv. Included articles were limited to RCTs on COVID-19 vaccines. A total of 73,633 subjects from 14 articles were included to compare the risks of adverse events following immunization (AEFI) after vaccinating different COVID-19 vaccines. Pooled risk ratios (*RR*) of total AEFI for inactivated vaccine, viral-vectored vaccine, and mRNA vaccine were 1.34 [95% confidence interval (*CI*) 1.11–1.61, *P* < 0.001], 1.65 (95% *CI* 1.31–2.07, *P* < 0.001), and 2.01 (95% *CI* 1.78–2.26, *P* < 0.001), respectively. No significant differences on local and systemic AEFI were found between the first dose and second dose. In addition, people aged ≤ 55 years were at significantly higher risk of AEFI than people aged ≥ 56 years, with a pooled *RR* of 1.25 (95% *CI* 1.15–1.35, *P* < 0.001).

**Conclusions:**

The safety and tolerance of current COVID-19 vaccine candidates are acceptable for mass vaccination, with inactivated COVID-19 vaccines candidates having the lowest reported AEFI. Long-term surveillance of vaccine safety is required, especially among elderly people with underlying medical conditions.

**Graphic Abstract:**

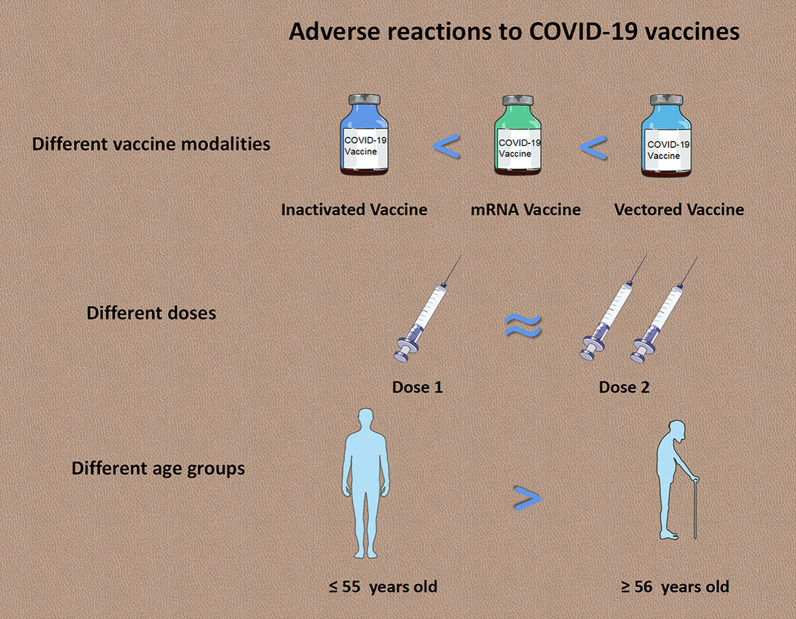

**Supplementary Information:**

The online version contains supplementary material available at 10.1186/s40249-021-00878-5.

## Background

The severe acute respiratory syndrome coronavirus 2 (SARS-CoV-2) infection has not been fully controlled yet, and the pandemic of coronavirus disease 2019 (COVID-19) continues to threaten the global public health. As of May 29, 2021, more than 170 million of infection cases and 3.5 million related deaths were confirmed. The numerous variants of SARS-CoV-2 strains are frequently emerging, which makes the situation more complicated, and the epidemic rebounds even in some countries/areas where it was initially controlled. More than 60 countries have discovered either community transmission or imported cases of variants strains [1]. Many interventions, including mask wearing, quarantining, and social distancing etc., have played important roles in controlling the spread of SARS-CoV-2 infection [2], but vaccination is generally thought as the most cost-effective intervention to eventually terminate the COVID-19 pandemic by establishing herd immunity among general population [3].

During the past 1 year, clinically-available COVID-19 vaccines have been developed at an unprecedented speed. According to the latest data of World Health Organization (WHO), at least 10 kinds of COVID-19 vaccines based on multiple technologies, represented by inactivated vaccine, viral vector vaccine and mRNA vaccine, have been approved for emergency clinical use or conditional marketing [4]. Russia firstly approved the use of viral vector COVID-19 vaccine on August 11, 2020 [5], followed by the United States, the United Kingdom, Canada and the European Union who successively approved the emergency use or marketing of Pfizer-BioNTech’s mRNA vaccine (BNT162b2) [6]. China has officially approved the conditional marketing of COVID-19 vaccine developed by Sinopharm’s China National Biotec Group (CNBG) since December 30, 2020 [7]. WHO also issued an emergency use authorization (EUA) of BNT162b2 vaccine on December 31, 2020 [8]. In addition, the United States issued an EUA for Moderna mRNA-1273 vaccine [9].

With COVID-19 vaccines for mass vaccination, one extremely important prerequisite is to illustrate their safety with confirmed clinical evidences. Vaccine hesitancy, which refers to the delay in acceptance or refusal of available vaccination, is a common public problem in the application and promotion of various vaccines [10–13]. In particular, the accelerated development process of COVID-19 vaccines might raise more concerns regarding their potential safety problems, and thereby deepen the vaccine hesitancy among people [14]. An increasing number of clinical trials assessing safety of COVID-19 vaccines are being published. A systematic review is urgently needed to provide a better understanding on safety of these vaccines. In order to better inform COVID-19 vaccination policies and reduce people’s vaccine hesitancy, we conducted a systematic review and meta-analysis to determine the safety of existing COVID-19 vaccine candidates from randomized controlled trials (RCTs).

## Method

### Search strategy and selection criteria

Our systematic review and meta-analysis followed the Preferred Reporting Items for Systematic Reviews and Meta-Analyses (PRISMA) guidelines [15]. We searched for literature published before March 3, 2021 in PubMed, Embase, Web of Science, the Cochrane library and Scopus, using the following search terms “(COVID-19 OR SARS-CoV-2 OR 2019-nCoV) AND (vaccin*) AND (safety OR adverse event* OR tolerance)”. We also retrieved any potentially related publications in the preprint database MedRxiv. The search was limited in English language papers. Reference management was performed in Endnote X9 (Clarivate Analytics, USA).

Only RCT studies evaluating the safety of COVID-19 vaccines were included. Eligible studies should meet the following criteria: blinding was involved; safety of both vaccination and control groups was reported; data on solicited local and systemic reactions during the first seven days, any injection local adverse reactions (such as pain, itching, redness, swelling, and induration, etc.) and general adverse reactions (such as cough, diarrhea, fatigue, fever, and headache, etc.) after vaccination, were available. Studies on all COVID-19 vaccines were included regardless of dosage form, schedule, preparation, or route of administration. Literature without original data on safety of COVID-19 vaccines among humans, including reviews, editorials, letters, animal studies, case reports, and comments, was excluded. Conference abstracts and studies without detailed AEFI data were excluded.

### Data extraction

Two researchers (MC and ZD) extracted data independently, and discrepancies were resolved through discussion with a third experienced one. The following data were extracted when available: first author, time of publication, characteristics of study subjects (age, number, etc.), intervention measures (vaccine type, number of doses, immunological dosage, adjuvant addition and adjuvant type, etc.), incidence of AEFI, and trial design. If some data were not available, the required data were calculated from the percentages reported in the study accordingly.

### Quality assessment

According to the assessment criteria of Cochrane Risk of Bias tool for RCTs, the methodological quality evaluation of included trials was carried out independently by two researchers (YY and ZD). The evaluation criteria included: random sequence generation, allocation concealment, blinding of participants and personnel, blinding of outcome assessment, incomplete outcome data, selective reporting and other biases. The quality of studies was determined according to: five or more items were at low risk of bias, three to four items were at moderate risk of biases, and less than three items were at high risk of bias.

### Data synthesis and analysis

We pooled the incidence of total AEFI in the vaccination group, and then compared the risks of AEFI (including total adverse reactions, any systemic and local adverse reactions, and single adverse reactions) between the vaccination group and the placebo group to assess the safety of vaccination. High heterogeneity was assumed among included studies with different study designs, and thus a random-effects model was used to calculate pooled effect sizes. The main indicators used were risk ratios (*RR*s) and their 95% confidence intervals (*CI*s) to report the risk of AEFI in the vaccination group relative to the control group. *RR* > 1 represented a risk effect. The *I*^2^ statistic was used to assess the level of statistical heterogeneity (*I*^2^ < 25.0%, low heterogeneity; 25.0–75.0%, moderate heterogeneity; and > 75.0%, considerable heterogeneity). We did subgroup analyses on AEFI according to the following potential sources of heterogeneity: vaccine types (inactivated vaccines, viral vector vaccines and mRNA vaccines, and subunit vaccine was excluded because of only one article available), sample sizes (large sample: *n* ≥ 500; small sample: *n* < 500), and trial phases (phase I, II, and III). In the current COVID-19 vaccine immunization strategies, two or more doses are usually needed to achieve an ideal protection efficacy, and we therefore further compared the AEFI incidence between the priming dose and the booster dose in some studies (dose1 vs. dose2), as well as the AEFI incidence between two age groups (≤ 55 years vs. ≥ 56 years). We did all analyses using STATA version 16.0 (College Station, Texas, USA) and Review Manager version 5.3 (Copenhagen: The Nordic Cochrane Centre, The Cochrane Collaboration). The statistic *P* < 0.05 was considered to be statistically significant in this meta-analysis.

## Results

### Characteristics of included studies

**Fig. 1:**
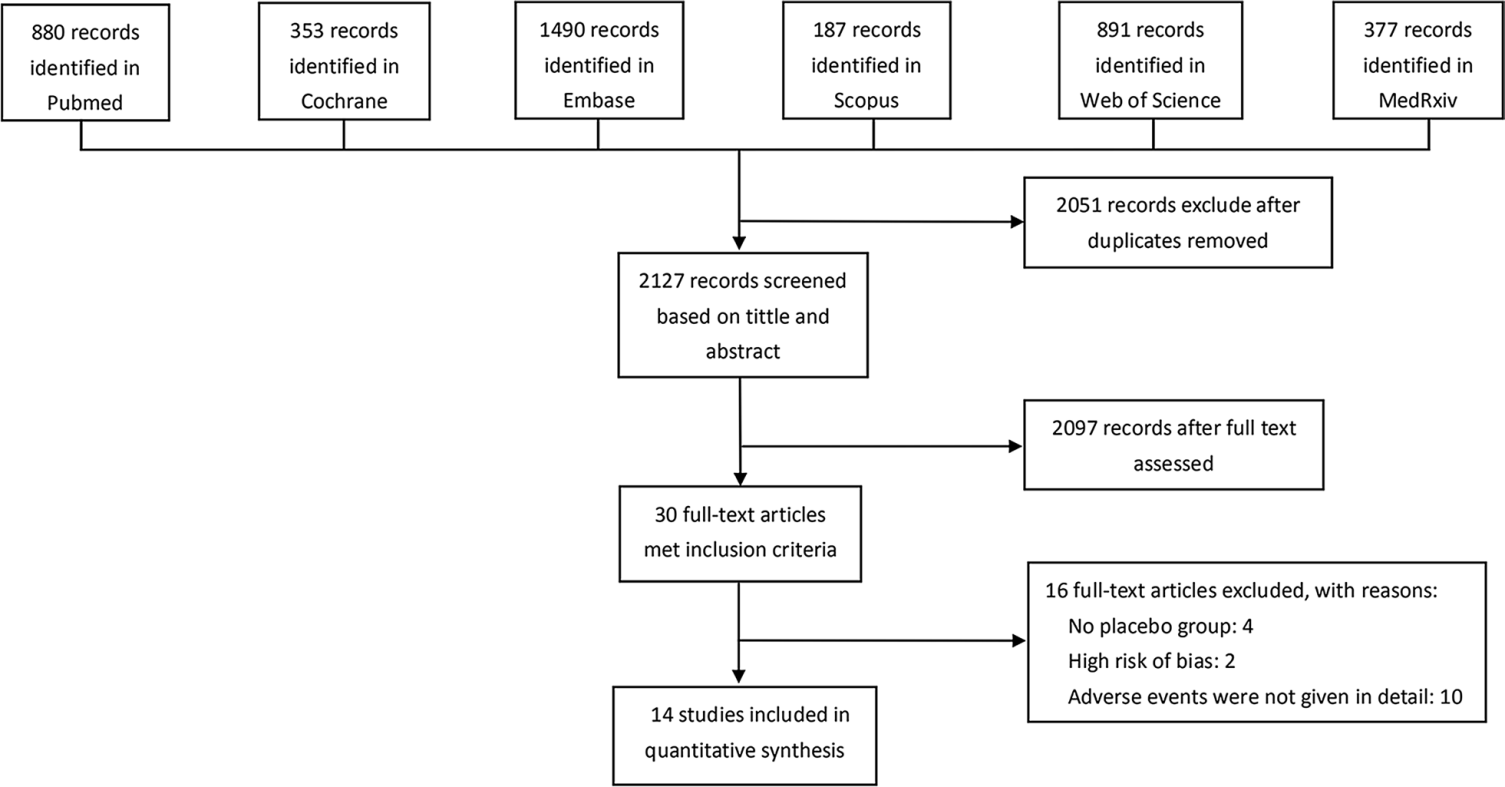
Selection of reports for inclusion in this systematic review

The flowchart of literature screening in this study is shown in Fig. 1. In our preliminary search, we got 4178 records from 6 databases. According to above eligible criteria, 14 studies (73,633 subjects) were finally included in this meta-analysis [16–29]. Of these, 13 studies were officially published, and one study was published on the medRxiv platform. Included studies contained ten kinds of COVID-19 vaccines, and they were further classified into four vaccine types on the basis of different technology platforms: inactivated vaccines [25–29], viral vector vaccines [22–24], subunit vaccines [21] and mRNA vaccines [16–20]. Among them, six studies were conducted in China [24–29]; three studies in the United States [16, 18, 20]; two in the United Kingdom [22, 23]; one in Germany and Belgium [17], one in Australia [21], and one from multiple-centers including the United States, Argentina, Brazil, Germany, South Africa and Turkey[19]. There were six reports on phase I trial [17, 20, 21, 27–29], five reports on phase II trial [24, 25, 27–29], and two reports on phase III trial [16, 19]. In addition, three reports were on phase I/II trial [19, 22, 26], and one on phase II/III trial [23]. The basic characteristics of the included RCTs are described in Additional file 1: Table S1.

Among the 14 included studies, ten reported the incidence of total adverse reactions, seven reported the incidence of systemic and local adverse reactions, and 13 studies reported the incidence of single adverse reactions such as pain and fever. All participants were over 16 years old, and four studies reported the AEFI incidence in different age groups (three studies were grouped by age ≤ 55 years old and ≥ 56 years old [19, 20, 23]).

We performed the quality assessment for included studies, and their bias risks are shown in Additional file 1: Figure S1, dominated by attrition bias and reporting bias. In some studies, particularly phase III clinical trials, incomplete data due to failure in follow-up led to high risk of attrition bias. Low risks of selection, performance and detection biases were found as a result of the appropriate implementation of these RCTs. In summary, 12 studies were at low risk of bias and two studies were at moderate risk of bias. Evidence of publication bias was found in studies reporting the total adverse reactions (asymmetrical funnel plot and *P* = 0.005 by Egger’s test; Additional file 1: Figure S17).

### Safety of COVID-19 vaccines

#### Adverse reactions to different COVID-19 vaccines

**Fig. 2:**
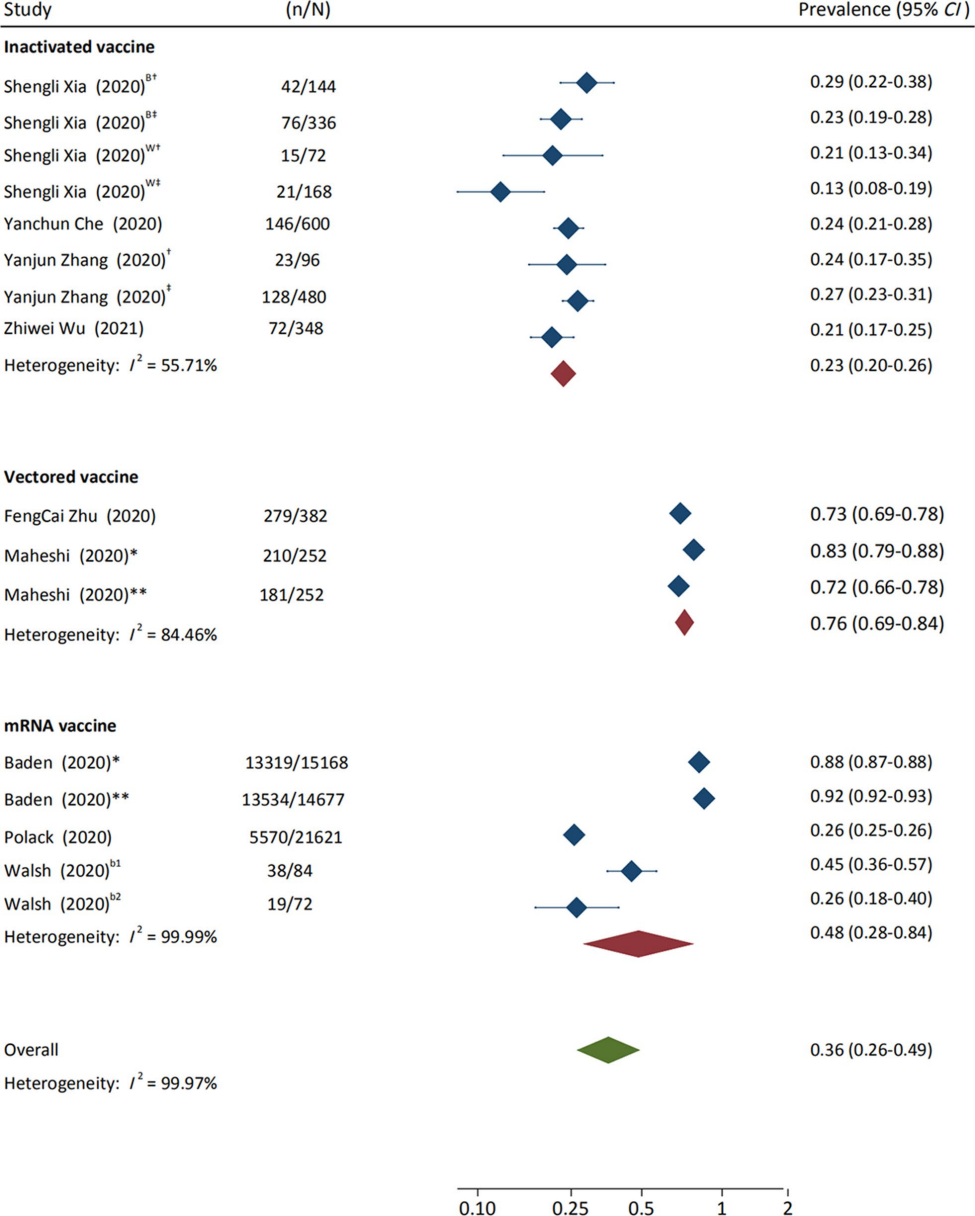
Incidence of adverse events following immunization for different vaccine modalities. ^B^BBIBP-CorV designed by the Beijing Institute of Biological Products; ^W^CoronaVac designed by the Wuhan Institute of Biological Products; ^†^COVID-19 vaccines in the Phase I Trial; ^‡^COVID-19 vaccines in the Phase II Trial; *COVID-19 vaccines on first vaccination; **COVID-19 vaccines on second vaccination; ^b1^BNT162b1 in the Phase I Trial; ^b2^BNT162b2 in the Phase I Trial. *RR* risk ratio, *CI* confidence interval, *mRNA* messenger ribonucleic acid

The AEFI incidence from ten studies was pooled, which contained 14 clinical trials with different COVID-19 vaccines and immunization procedures. We found that the pooled AEFI incidence of inactivated vaccines, mRNA-based vaccines and viral-vector vaccines was 23.0% (95% *CI* 20.0–26.0%, *I*^2^ = 55.71%), 48.0% (95% *CI* 28.0–84.0%, *I*^2^ = 99.99%), 76.0% (95% *CI* 69.0–84.0%, *I*^2^ = 84.46%), respectively (Fig. 2).

We compared the AEFI occurrences of different vaccine modalities between vaccination group and control group. The pooled *RR*s of total adverse reactions (*RR* = 1.75, 95% *CI* 1.59–1.92), systemic adverse reactions (*RR* = 1.41, 95% *CI* 1.11–1.78), and local adverse reactions (*RR* = 4.49, 95% *CI* 3.79–5.30) for all vaccines were significantly higher in the vaccination group, but the heterogeneity among these meta-analyses was considerable (*I*^2^ = 92.76%, 99.09%, and 93.86%, respectively) (Table 1 and Additional file 1: Figure S2–S4). We subsequently performed subgroup meta-analysis for four vaccine types. Of note, we found that the heterogeneity of the analysis on total adverse reactions (*RR* = 1.34, 95% *CI* 1.11–1.61), systemic adverse reactions (*RR* = 0.92, 95% *CI* 0.69–1.23) and local adverse reactions (*RR* = 1.94, 95% *CI* 1.10–3.41) for the group of inactivated vaccines was greatly reduced to an extremely low level (*I*^2^ = 0.00%, 0.00%, and 54.18%, respectively) compared to results of all the previous analyses. Pooled RRs of systemic adverse reactions to the inactivated vaccines, such as fever (*RR* = 0.99, 95% *CI* 0.56–1.73, *I*^2^ = 0.00%), headache (*RR* = 0.72, 95% *CI* 0.29–1.77, *I*^2^ = 0.00%), fatigue (*RR* = 0.85, 95% *CI* 0.55–1.30, *I*^2^ = 0.00%), were of no significant difference from that in the control group. In addition, pooled RRs of local adverse reactions to the inactivated vaccines, such as pain (*RR* = 2.24, 95% *CI* 1.37–3.65, *I*^2^ = 50.44%), redness (*RR* = 0.90, 95% *CI* 0.32–2.59, *I*^2^ = 0.00%), swelling (*RR* = 1.08, 95% *CI* 0.39–3.03, *I*^2^ = 0.00%) were slightly higher than or similar to that in the control group (Table 1 and Additional file 1: Figures S5–S8).

**Table 1:** Incidence of total adverse reactions among vaccination group versus control group

	No. of studies	Reactions/total		*RR* (95% *CI*)	*I*^2^
		Vaccination	Control		
Overall					
Total adverse reactions	10	33 673/54 752	16 446/52 400	1.75 (1.59–1.92)*	0.93
Systemic adverse reactions (any)	7	25 286/39 698	14 972/38 088	1.41 (1.11–1.78)*	0.99
Local adverse reactions (any)	7	32 077/39 698	6687/38 088	4.49 (3.79–5.30)*	0.94
Total adverse reactions to different vaccine types					
Inactivated vaccine	5	523/2244	107/630	1.34 (1.11–1.61)*	0.00
Vectored vaccine	2	670/886	177/382	1.65 (1.31–2.07)*	0.75
mRNA vaccine	3	32 480/51 622	16 162/51 388	2.01 (1.78–2.26)*	0.98
Systemic adverse reactions (any) to different vaccine types					
Inactivated vaccine	4	193/1764	52/468	0.92 (0.69–1.23)	0.00
mRNA vaccine	2	25 020/37 696	14 911/37 560	1.65 (1.21–2.24)*	1.00
Local adverse reactions (any) to different vaccine types					
Inactivated vaccine	4	172/1164	29/320	1.94 (1.10–3.41)*	0.54
mRNA vaccine	2	31 735/37 696	6653/37 560	5.37 (4.54–6.36)*	0.98
Fever					
Inactivated vaccine	5	64/2244	16/630	0.99 (0.56–1.73)	0.00
Vectored vaccine	3	410/1121	80/968	3.05 (1.56–5.99)*	0.84
mRNA vaccine	5	3175/38 365	128/37 591	7.90 (2.72–22.94)*	0.95
Headache					
Inactivated vaccine	4	22/1644	6/480	0.72 (0.29–1.77)	0.00
Vectored vaccine	3	555/1121	311/968	1.68 (1.51–1.86)*	0.00
mRNA vaccine	5	17 152/38 365	9300/37 591	2.06 (1.49–2.83)*	0.99
Fatigue					
Inactivated vaccine	5	88/2244	6/630	0.85 (0.55–1.30)	0.00
Vectored vaccine	3	639/1121	334/968	1.88 (1.31–2.69)*	0.81
mRNA vaccine	5	19 471/38 365	9506/37 591	1.98 (1.46–2.67)*	0.99
Pain					
Inactivated vaccine	5	329/2244	39/630	2.24 (1.37–3.65)*	0.50
Vectored vaccine	2	573/925	209/660	3.29 (0.92–11.77)	0.95
mRNA vaccine	4	31 924/38 065	5946/37 519	5.63 (4.89–6.48)*	0.93
Redness					
Inactivated vaccine	5	15/2244	2/630	0.90 (0.32–2.59)	0.00
Vectored vaccine	2	22/925	13/660	1.34 (0.67–2.65)	0.00
mRNA vaccine	3	2127/37 650	194/37 457	7.59 (3.74–15.39)*	0.93
Swelling					
Inactivated vaccine	4	19/1644	3/480	1.08 (0.39–3.03)	0.00
Vectored vaccine	2	37/925	18/660	2.23 (0.33–14.96)	0.53
mRNA vaccine	4	3843/38 065	149/37 519	12.69 (6.50–24.79)*	0.89
Total adverse reactions of different sample sizes					
Sample size ≥ 500	5	32 976/52 928	16 246/51 758	1.95 (1.75–2.17)*	0.97
Sample size < 500	6	697/1824	200/652	1.43 (1.25–1.64)*	0.12
Total adverse reactions of different trial phases					
Phase I trial	4	137/468	26/155	1.62 (1.12–2.36)*	0.00
Phase II trial	5	650/1966	120/564	1.49 (1.14–1.94)*	0.54
Phase III trial	2	32 423/51 466	16 154/51 352	2.03 (1.79–2.29)*	0.99

*RR* risk ratios, *CI* confidence interval, *No.* Number

^*^P < 0.05

We observed high heterogeneity in the meta-analyses of total adverse reactions (*RR* = 2.01, 95% *CI* 1.78–2.26, *I*^2^ = 97.55%), systemic adverse reactions (*RR* = 1.65, 95% *CI* 1.21–2.24, *I*^2^ = 99.75%), and local adverse reactions (*RR* = 5.37, 95% *CI* 4.54–6.36, *I*^2^ = 97.85%) for the group of mRNA vaccines (Table 1 and Additional file 1: Figures S2, S5, S6). Pooled *RR*s of systemic adverse reactions to mRNA vaccines, such as fever (*RR* = 7.90, 95% *CI* 2.72–22.94, *I*^2^ = 94.88%), headache (*RR* = 2.06, 95% *CI* 1.49–2.83, *I*^2^ = 98.92%), fatigue (*RR* = 1.98, 95% *CI* 1.46–2.67, *I*^2^ = 99.04%), were significantly higher than that in the control group. Similar differences were observed on single local adverse reactions such as pain (*RR* = 5.63, 95% *CI* 4.89–6.48, *I*^2^ = 92.71%), redness (*RR* = 7.59, 95% *CI* 3.74–15.39, *I*^2^ = 93.31%), swelling (*RR* = 12.69, 95% *CI* 6.50–24.79, *I*^2^ = 88.65%) (Table 1 and Additional file 1: Figures S9, S10).

Due to insufficient data on viral-vector vaccines, only total adverse reactions were pooled (*RR* = 1.65, 95% *CI* 1.31–2.07) with a moderate heterogeneity (*I*^2^ = 75.07%) (Table 1 and Additional file 1: Figure S2). Incidence of single systemic adverse reactions to the viral-vector vaccines, such as fever (*RR* = 3.05, 95% *CI* 1.56–5.99, *I*^2^ = 83.86%), headache (*RR* = 1.68, 95% *CI* 1.51–1.86, *I*^2^ = 0.00%) and fatigue (*RR* = 1.88, 95% *CI* 1.31–2.69, *I*^2^ = 80.52%), was significantly higher than that in the control group. In addition, incidence of single local adverse reactions to the viral-vector vaccines, such as pain (*RR* = 3.29, 95% *CI* 0.92–11.77, *I*^2^ = 94.75%), redness (*RR* = 1.34, 95% *CI* 0.67–2.65, *I*^2^ 0.00%), swelling (*RR* = 2.23, 95% *CI* 0.33–14.96, *I*^2^ = 53.29%) was of no difference from that in the control group (Table 1 and Additional file 1: Figures S11, S12).

We conducted subgroup analysis on studies with large sample size (≥ 500) and small sample size (< 500). Heterogeneity of the small sample size meta-analysis was significantly lower than that with large sample size (*I*^2^ = 12.25% vs *I*^2^ = 97.02%) (Table 1 and Additional file 1: Figure S13). We found zero to moderate heterogeneity among studies in phase I (*I*^2^ = 0.00%), phase II (*I*^2^ = 53.96%), and considerable heterogeneity (*I*^2^ = 98.77%) in phase III (Table 1 and Additional file 1: Figure S14). Heterogeneity among studies in phase I was significantly lower than that in phase II and phase III, consistent with above observation in the subgroup analysis on studies with different sample sizes.

#### Adverse reactions to different inoculation doses

**Fig. 3:**
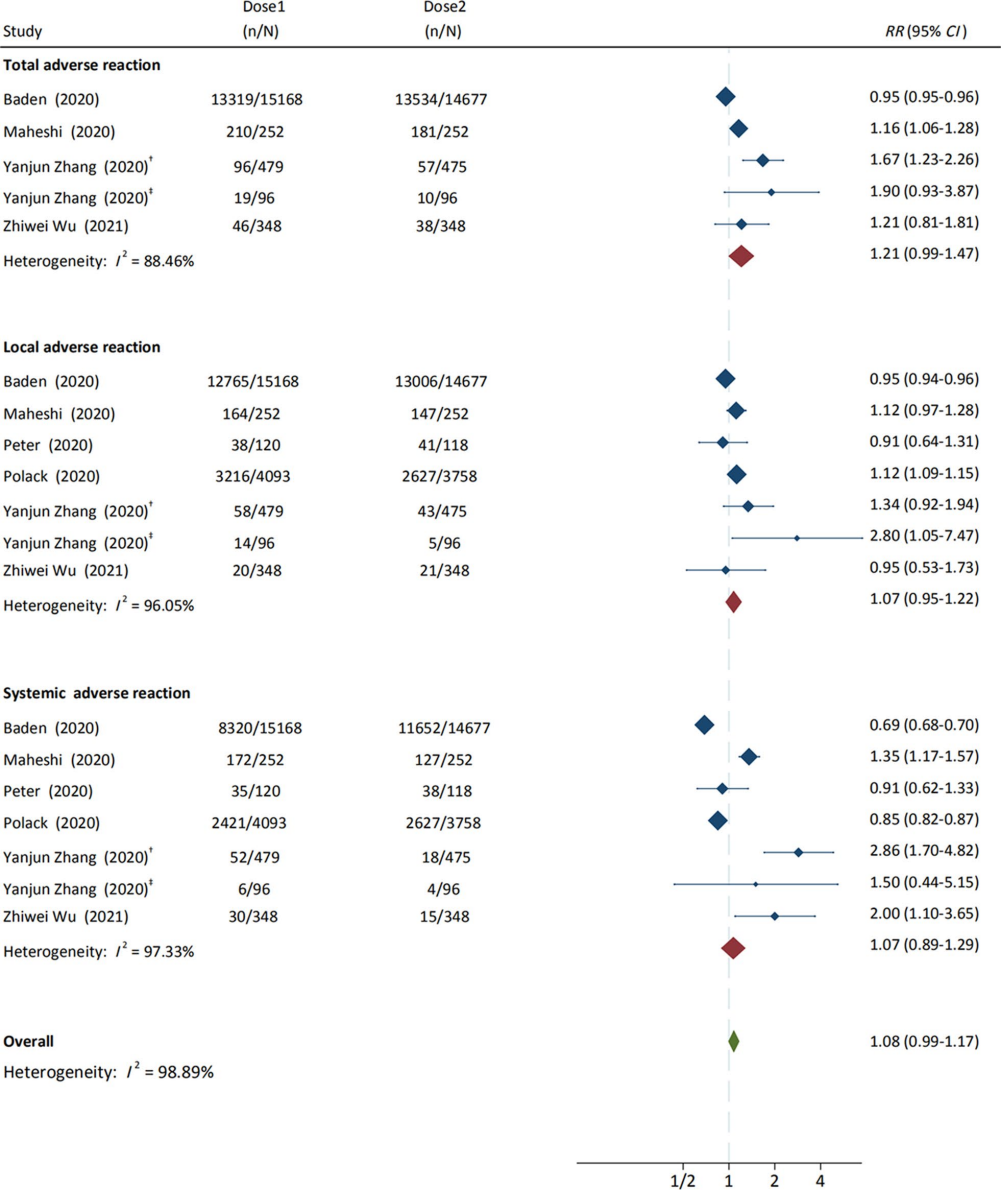
Adverse reactions to the first dose and second dose of COVID-19 vaccines. ^†^COVID-19 vaccines in the Phase I Trial; ^‡^COVID-19 vaccines in the Phase II Trial. *CI* confidence interval, *mRNA* messenger ribonucleic acid

Results showed that the pooled RRs of total adverse reactions (*RR* = 1.21, 95% *CI* 0.99–1.47, *I*^2^ = 88.46%), systemic adverse reactions (*RR* = 1.07, 95% *CI* 0.89–1.29, *I*^2^ = 97.33%), and local adverse reactions (*RR* = 1.07, 95% *CI* 0.95–1.22, *I*^2^ = 96.05%) to the first dose, were of no significant difference from that to the second dose (Fig. 3).

### Adverse reactions in different age groups

**Fig. 4:**
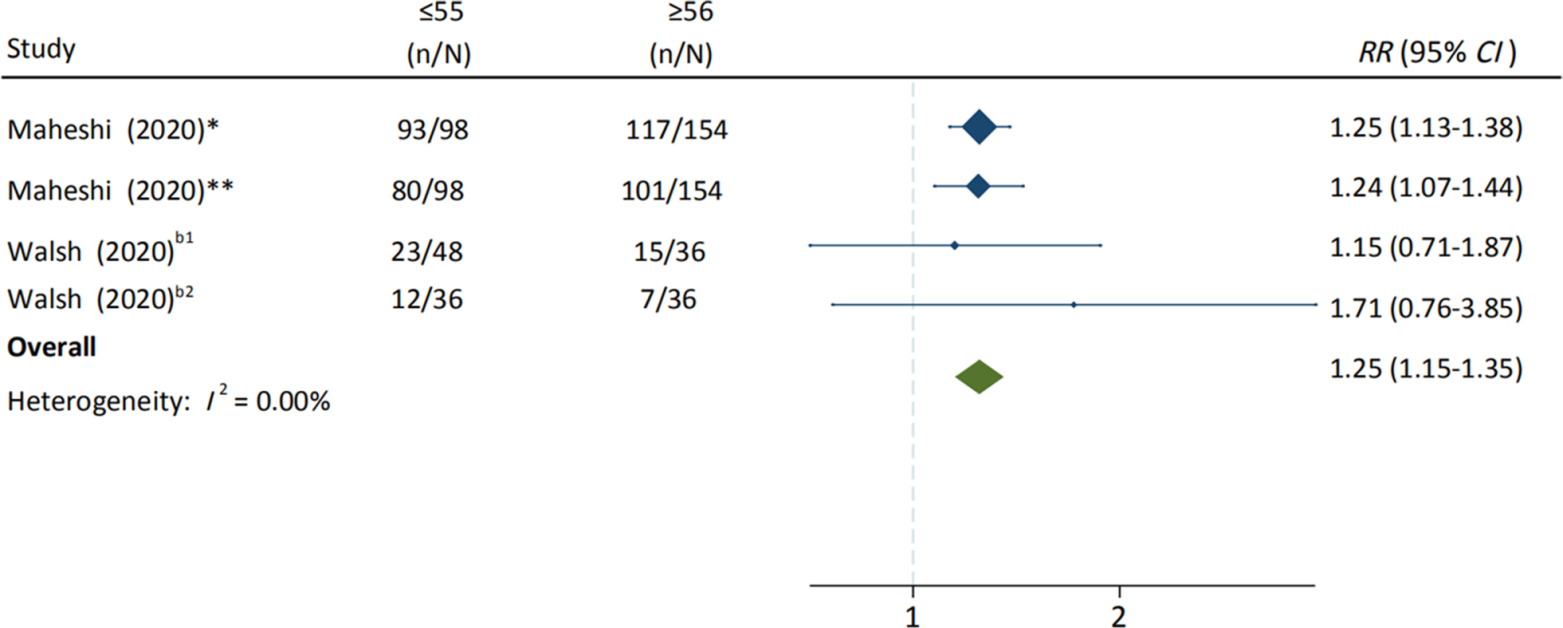
Adverse reactions to COVID-19 vaccines between population aged ≤ 55 years and population aged ≥ 56 years. *COVID-19 vaccines on first vaccination; **COVID-19 vaccines on second vaccination; ^b1^BNT162b1 in the Phase I Trial; ^b2^BNT162b2 in the Phase I Trial. *CI* confidence interval, *mRNA* messenger ribonucleic acid

We found that the risk of total AEFI in young people (≤ 55 years old) was significantly higher than that in the elderly (≥ 56 years old) (*RR* = 1.25, 95% *CI* 1.15–1.35), and no heterogeneity was found (Fig. 4). Pooled RRs of systemic adverse reactions in population aged ≤ 55 years, such as fever (*RR* = 1.83, 95% *CI* 1.30–2.57, *I*^2^ = 51.26%) and fatigue (*RR* = 1.41, 95% *CI* 1.26–1.59, *I*^2^ = 61.97%), were significantly higher than that in population aged ≥ 56 years. In addition, pooled RRs of local adverse reactions in population aged ≤ 55 years, such as pain (*RR* = 1.31, 95% *CI* 1.17–1.45, *I*^2^ = 85.95%), redness (*RR* = 0.89, 95% *CI* 0.74–1.07, *I*^2^ = 0.00%), and swelling (*RR* = 0.87, 95% *CI* 0.74–1.03, *I*^2^ = 0.00%) were slightly higher than or similar to that in population aged ≥ 56 years. (Additional file 1: Figures S15, S16).

## Discussion

Our study found inactivated vaccines had much lower AEFI incidence than viral vector vaccines and mRNA vaccines. Mild local reactions including pain, swelling and/or redness at the injection site were common/very common after vaccination against COVID-19, which is similar to other injectable vaccines such as whole-cell pertussis vaccine or human papillomavirus (HPV) vaccine [10, 30]. The occurrence of systemic reactions varies in response to different antigen immunization. Fever can occur in about 10% or more of vaccinees [10]. Other mild systemic reactions (e.g., headache, fatigue) are also common to occur after vaccination. For example, after immunization with bivalent HPV vaccine, the occurrence of fatigue and headache can be up to 33.0% and 30.0%, respectively [10].

An ideal vaccine is expected to induce a protective immunity against specific pathogens without any adverse reaction. However, in clinical application, a certain probability of side effects happens by chance. AEFI is any untoward medical occurrence following immunization, which does not necessarily have a causal relationship with vaccination. In most cases, the exact mechanism of the adverse reactions by vaccination is unclear, but it might be related to non-specific immune responses by the components of vaccines (e.g., adjuvant, stabilizers or preservatives)*.* AEFI may be any unfavorable or unintended sign, abnormal laboratory finding, symptom or disease [10], including local adverse reactions, such as injection site pain, redness, swelling, etc., and systemic adverse reactions, such as fever, headache, fatigue, nausea, vomiting, diarrhea, etc. In general, similar to other traditional vaccines, AEFI symptoms of COVID-19 vaccines are mild. Severe adverse events (SAEs) were rare, and only were reported in some volunteers as hypersensitivity, facioplegia, urticaria and anaphylactic shock [11]. Vaccine-associated anaphylaxis was rare, approximately one case per million injections, for most known vaccines [31]. Of note, the incidence of anaphylaxis associated with the Pfizer COVID-19 mRNA vaccine was reported to be approximately ten times as high as that reported in all previous vaccines [11]. The Pfizer-BioNtech and Moderna vaccines were the first mRNA vaccine modalities to obtain an EUA in vaccinating healthy people, however, the mechanism of allergic reactions associated with mRNA vaccines is still unclear. It is possible that some people are at a higher risk for non-IgE-mediated mast-cell activation or complement activation related to either the lipid or the polyethylene glycol (PEG)-lipid component of the vaccine [32]. According to the current recommendations, people with a history of an anaphylactic reaction to any component of the mRNA COVID-19 vaccines should avoid taking mRNA vaccines, and this recommendation would currently exclude people with a history of immediate reactions associated with PEG [33]. Very few cases of Bell’s palsy following vaccination were reported in clinical trials on both the Pfizer-BioNTech vaccines and Moderna vaccines [16, 19]. However, no evidence showed these cases were causally related to vaccination. Ongoing surveillance of long-term safety of mRNA vaccines is needed [33].

No significant correlation between the number of inoculation doses and AEFI occurrence was found, which is consistent with the previous literature on other vaccines [34, 35]. Among the currently marketed COVID-19 vaccines, only CanSino Biologics and Johnson viral-vector vaccines adopted single-needle immunization schedule. Single-dose vaccination is more feasible for mass population and contributes to a higher acceptance of vaccination. However, our data showed that for most vaccines, multi-doses vaccination had better immunogenicity and efficacy without increasing side effects, which might help to decrease the vaccine hesitancy for multi-doses vaccination. As the COVID-19 pandemic is raging on in many parts of the world, the public should be vaccinated as soon as possible to build the herd immunity against SARS-CoV-2.

The ability in eliciting immune responses to vaccination usually varies by age. Considering that the elderly are susceptible to SARS-CoV-2 infection [36], it is worthy to pay attention to the safety of vaccine in this population. The overall AEFI incidence was higher for vaccinees aged 16–55 years than that among elderly people, which is consistent with the previous studies [37, 38]. In both trivalent influenza vaccine (TIV) and tetravalent influenza vaccine (QIV), older adults showed lower systemic reaction rates than younger adults [39]. With aging, the function of the immune system declines, a phenomenon also referred to as immunosenescence [40]. Profound changes of the immune system include the gradual loss of naive cells, increase of memory cell numbers, and decrease in the diversity of T cell and B cell repertoire [41, 42]. These changes lead to reduced protection against infectious diseases and reduced vaccine responses in older adults. Consequently, in response to immunization, both inflammatory reactions and protective immune responses in elderly population are slower, weaker and more transitory than that in younger healthy adults [36]. However, the molecular mechanisms underlying age-related hyporesponsiveness to vaccination remain unclear [43]. Data for the first month after mass vaccination against COVID-19 in the United States indicated that 150 (2.1%) AEFI cases were reported among long-term care facility (LTCF) residents who were vaccinated at an average age of 83 (range: 17–104) years. Among 122 (81.3%) cases of SAE in LTCFs, 78 (52.0%) deaths were reported [44]. Furthermore, since the start of vaccination project at the end of 2020, Norway has reported 33 deaths among elderly people after their first dose, but no evidence showed the link between COVID-19 vaccination and these deaths [45]. It is worth noting that the patients with SAEs or deaths in Norway mainly aged between 80 and 89 years. There was no evidence to prove the safety of vaccination in people aged over 85 years. In the absence of sufficient evidence to show the safety of COVID-19 candidate vaccines for elderly people, elderly people with underlying medical conditions might be cautious to get vaccination.

High speed of COVID-19 vaccine development and deployment has led to numerous concerns in the public about the safety of these new vaccines. Some media failed to report information on COVID-19 vaccines accurately and scientifically, which might deepen vaccine hesitation in the public and impede mass immunization. In response to these concerns, spontaneous (or passive) immunization safety surveillance systems, such as the US Vaccine Adverse Event Reporting System (VAERS) and China National AEFI Information System (CNAEFIS) [46], were launched at national or international levels to ensure effective monitoring and prompt actions in response to AEFIs after COVID-19 vaccination.

The results of our study should be interpreted with caution because it has some limitations. First, although we tried to decrease some heterogeneity by doing subgroup analyses on many trial characteristics, high statistical heterogeneity existed for some effect sizes, which might be due to the diversity in the schedule of vaccination, follow-up, vaccine component, and study populations. For example, high heterogeneity was found in the pooled estimates of the total AEFI. After we conducted subgroup analyses by vaccine type, heterogeneity significantly decreased, suggesting that vaccine type might be a source of heterogeneity in pooling estimates of AEFI. Second, we failed to make a subgroup analysis on vaccination doses due to varied doses for different vaccines. Third, in most trials, AEFI was usually recorded within 7–10 days after vaccination, with limited sample size. Even data from the phase III clinical trials, which provided important evidence to illustrate the safety of COVID-19 vaccines, are limited by short-term follow-up. It is necessary to perform long-term surveillance of vaccine safety in large-scale population [47].

In addition, we just focused on the safety of current vaccines in this study, which might lead to the one-sidedness in a comprehensive evaluation of COVID-19 vaccines since we have ignored their efficacy. So far, more than 1 billion people worldwide have been immunized with different COVID-19 vaccines worldwide. Mass vaccination with highly efficacious vaccines is critical to establish a herd immunity to stop the COVID-19 pandemic. Although current published data of vaccine effectiveness is not enough to perform a meta-analysis yet, the vaccination has shown a good protection efficacy (50–90%). It is worth noting that, with fast-changing situation in the present pandemic, the public should get vaccinated as soon as the COVID-19 vaccine available, and then accomplish a population immune barrier against SARS-CoV-2 infection. Further analysis of vaccine efficacy should be done when enough literature data are available.

## Conclusions

Our findings contribute to understanding the profiles of existing COVID-19 vaccines and help policymakers to decide vaccination strategies. The safety of current COVID-19 vaccine candidates is acceptable for mass vaccination, with inactivated COVID-19 vaccines candidates having the lowest AEFI. The immunization safety surveillance systems worldwide should continue to monitor the long-term safety of marketed COVID-19 vaccines, especially among elderly people with underlying medical conditions, to inform vaccination policy and to maintain public confidence in COVID-19 vaccination.

## Supplementary Information

**Additional file 1.** Additional Figures S1–S17 and Table S1.

## Data Availability

All data generated or analyzed during this study are included in this published article and its additional information files.

## Abbreviations

RCTs: Randomized controlled trials
AEFI: Adverse events following immunization
COVID-19: Coronavirus disease 2019
*RR*: Risk ratio
*CI*: Confidence interval
SARS-CoV-2: Severe acute respiratory syndrome coronavirus 2
WHO: World Health Organization
CNBG: China National Biotec Group
PRISMA: Preferred Reporting Items for Systematic Reviews and Meta-Analyses
HPV: Human papillomavirus
PEG: Polyethylene glycol
LTCF: Long-term care facility
VAERS: Vaccine Adverse Event Reporting System
CNAEFIS: China National AEFI Information System
SAE: Severe adverse events
