# Supplementary material for: Safety of SARS-CoV-2 vaccines: a systematic review and meta-analysis of randomized controlled trials
Source: Infect Dis Poverty. 2021 Jul 5;10:94. doi: 10.1186/s40249-021-00878-5 (PMC8256217; doi:10.1186/s40249-021-00878-5)
Supplement: Supplementary file 1 — Additional file 1. Additional Figures S1–S17 and Table S1. [file 40249_2021_878_MOESM1_ESM.docx]

**Table S1. Basic characteristics of all included studies**

| **First author** | **Study design** | **Controlled/vaccinated  subjects** | **Subjects characteristics** | **Local reactions** | **Systemic reactions** | **Vaccine characteristics** | **Vaccination design** | |
| --- | --- | --- | --- | --- | --- | --- | --- | --- |
| **Inactivated vaccine** | | | | | | | | |
| Shengli Xia 2020 | randomized double-blind placebo-controlled phase I/II trials | 80/240 | Asian  mean age,42.8 years male, 37.5% | pain itching redness swelling | fever, coughing diarrhea, fatigue headache, pruritus nausea, vomiting | phase 1 trial: 2.5, 5, and 10 μ g/dose intramuscular injections; phase 2 trial: 5 μ g/dose in 2 schedule groups | phase I trial: 4,14, 21 days after each injection in first dose; 4 days and 14 days in second and third dose; phase II trial: 14 days after second dose | |
| Shengli Xia 2020 | randomized double-blind placebo-controlled studies phase I/II trial | 160/480 | Asian  mean age,45.3 years male, 45.7% | pain itch redness swelling rash | fever fatigue inappetence nausea diarrhoea joint pain Headache Vomiting | BBIBP-CorV  phase 1 trial: 2, 4, and 8 μ g/dose intramuscular injections; phase 2 trial: 4 μ g/dose in 3 schedule groups 8 μ g/dose in 1 schedule groups | phase I trial: 7,14, 28,32 and 42 days after injection; phase II trial: 14 and 28 days after injection 14 days after second dose | |
| Yanjun Zhang 2020 | double-blind randomised placebo-controlled phase I/II clinical trial | 167/576 | Han nationality  mean age,42.4 years male, 44.6% | pain swelling redness discoloration | fatigue diarrgoea fever muscle pain headache nausea cough hypersensitivity | CoronaVac  3 μ g/dose and 6 μ g/dose in 2 schedule groups; intramuscular injections | phase I trial: 7,14, 28 days after second injection; phase II trial: 14, 28 days after second injection in 0,14 schedule group 28 days after second injection in 0,28 schedule group | |
| Zhiwei Wu 2021 | double-blind randomised placebo-controlled phase I/II clinical trial | 73/498 | Han nationality  mean age,66.5 years male, 48.9% | pain swelling erythema pruritus | fatigue diarrgoea fever muscle pain headache nausea cough hypersensitivity | CoronaVac  phase 1 trial :3 μ g/dose and 6 μ g/dose;  phase 2 trial :1.5μ g/dose ,3 μ g/dose and 6 μ g/dose;  intramuscular injections | phase I trial: 7,14, 28 days after second injection; phase II trial: 14, 28 days after second injection in 0,14 schedule group 28 days after second injection in 0,28 schedule group | |
| Yanchun Che | double-blind randomised placebo-controlled phase II clinical trial | 150/600 | Han nationality  Mean age, 39.2 years  male, 37.7% | Pain  Redness  Swelling  Itch | Fever  Fatigue  Diarrhea  Hypersensitivity/urticaria  Cough  Nausea  Vomiting  Mucosal abnormality | Each 0·5 ml vaccine  dose, containing 100 or 150 EU inactivated viral antigen | record local and systemic reactions from 0 to 7 days, from 0 to 28 days after the booster immunization | |
| **Subunit vaccine** | | | | | | | | |
| Peter 2021 | randomised, double-blind, placebo-controlled  phase I | 30/120 | mean age, 45.8 years male,42.8% | pain  redness  swelling | Headache  fatigue  myalgia, nausea/vomiting, diarrhoea  vomiting | SCB-2019 recombinant SARS-CoV-2 trimeric S-protein subunit vaccine  Each 0·5 ml vaccine  dose, containing 3μg, 9μg or 30μg SCB-2019 | | record local and systemic reactions for 7 days blood samples were drawn on Days 8, 22, 36 and 50 |
| **Virus Vector Vaccine** | | | | | | | | |
| Fengcai Zhu 2020 | randomised double-blind placebo-controlled phase II trial | 126/382 | Asian  mean age,39.7 years male, 50.0% | pain swelling redness induration itch | fever, headache, fatigue, vomiting, diaeehoea, muscle pain, joint muscle, cough, nausea | Ad5-vectored COVID-19 vaccine  intramuscular injections; A single injection of the vaccination of 1 × 10¹¹ or 5 × 10¹⁰  viral particles per mL | 14, 28 days and 6 months after injection | |
| Pedro 2020 | participant-blinded; multicentre randomised controlled trial phase I/II | 534(MenACWY  group)/543 | median age,35 years male, 50.2% | pain swelling redness itch induration tenderness | chills, fatigue, fever, feverish, headache, joint pain, malaise, nausea, muscle ache | ChAdOx1 nCoV-19; chimpanzee  adenovirus-vectored vaccine  intramuscular injection: a dose of 5 × 10¹⁰ viral  particles, 10 participants received a booster  administration 28 days after the first dose. | | 3, 7, 14, 28, and 56 days after vaccination |
| Maheshi 2020 | single-blind,  randomised,  controlled, phase II/III trial | 140(MenACWY  group)/420 | median age, 18-55 years group: 43.0 years 56-69 years group: 60.0 years 70years and older: 73.0 years male,50.0% | pain swelling redness itch induration tenderness | chills, fatigue, fever, feverish, headache, joint pain, malaise, nausea, muscle ache | ChAdOx1 nCoV-19; chimpanzee  adenovirus-vectored vaccine  intramuscular injection: a lower dose of 2·2 × 10¹⁰ virus particles and a standard dose of 3·5–6·5 × 10¹⁰ virus  particles in one or two doses | | 0, 7, 14, and 28 days after their prime and booster vaccinations |
| **mRNA Vaccine** | | | | | | | | |
| Baden 2020 | phase III randomized observer-blinded placebo-controlled trial | 15179/15181 | mean age,52.4 years male,52.7% | Pain, Erythema, Swelling, Lymphadenopathy | Fever, Headache, Fatigue, Myalgia,  Arthralgia, Chills Nausea, Vomiting | mRNA-1273  intramuscular injections; 2 dose of mRNA-1273 (100 μg) | | 7 days and 28 days after each injection; monitoring adverse reactions   from day 1 through day 759 |
| Walsh 2020 | placebo-controlled, observer-blinded, randomized dose-escalation,  phase I trial | 21/84 | median age, BNT162b1: younger group,35 older group,69 BNT162b2: younger group,37 older group,68 male,44.8% | pain redness swelling | fever fatigue chills | BNT162b1 and BNT162b2  intramuscular injections; 10 μg, 20 μg,  or 30 μg of BNT162b1 or BNT162b2 (or placebo)  on a two-dose schedule; 100 μg for one younger group | | 7 days and 21 days after the first dose; 7 days and 14 days after the second dose |
| Mulligan 2020 | placebo-controlled,  observer-blinded, randomized, Phase I/II trial | 9/36 | mean age,35.4 years male,51.1% | pain redness swelling | fever, fatigue, chills, headache, vomiting, diarrhea, muscle pain, joint pain | BNT162b1  intramuscular injections; receive 2 doses,  separated by 21 days,  of 10 µg, 30 µg, or  100 µg of BNT162b1 | | 7,14,21,28,35 days after first dose |
| Polack 2020 | placebo-controlled,  observer-blinded, randomized,  Phase III trial | 18846/18860 | median age,52.0years male, 50.6% | pain redness swelling | fever, fatigue, chills, headache, vomiting, diarrhea, muscle pain, joint pain | BNT162b2  intramuscular injections; two 30-μg doses of BNT162b2; 21 days apart | | 7days after each dose; 1 and 6 months after second dose |
| Peter 2020 | placebo-controlled,  blinded, phase I trial | 32/216 | mean age,38.6 years male,57% | pain redness swelling itching | fever, fatigue, chills, headache, vomiting, diarrhea, myalgia | CvnCov; mRNA- Lipid Nanoparticle  Vaccine  intramuscular injections; 0.5ml of each dose (4, 6, 8, and 12 μg) | | 7 days and 28 days after vaccination;  Blood samples were also drawn on Days 1, 2, 8, 30 and 36 |

**Figure S1. Quality assessment for included studies.** a: Methodological quality of included studies. b: The distribution of the methodological quality of included studies.

a.


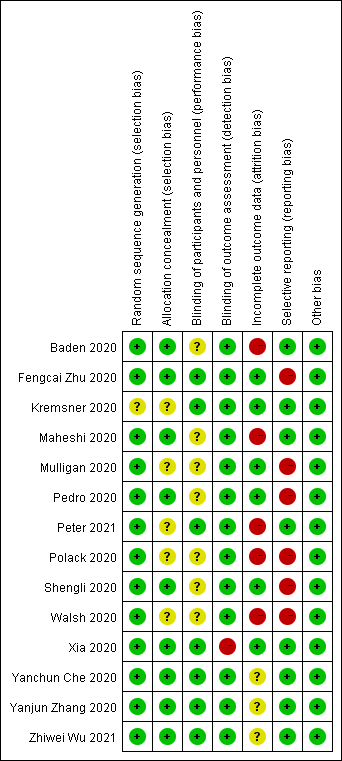


b.


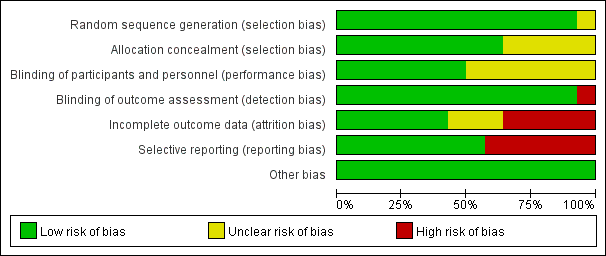


**Figure S2.** **Occurrence of total adverse reactions between vaccination group and placebo group**


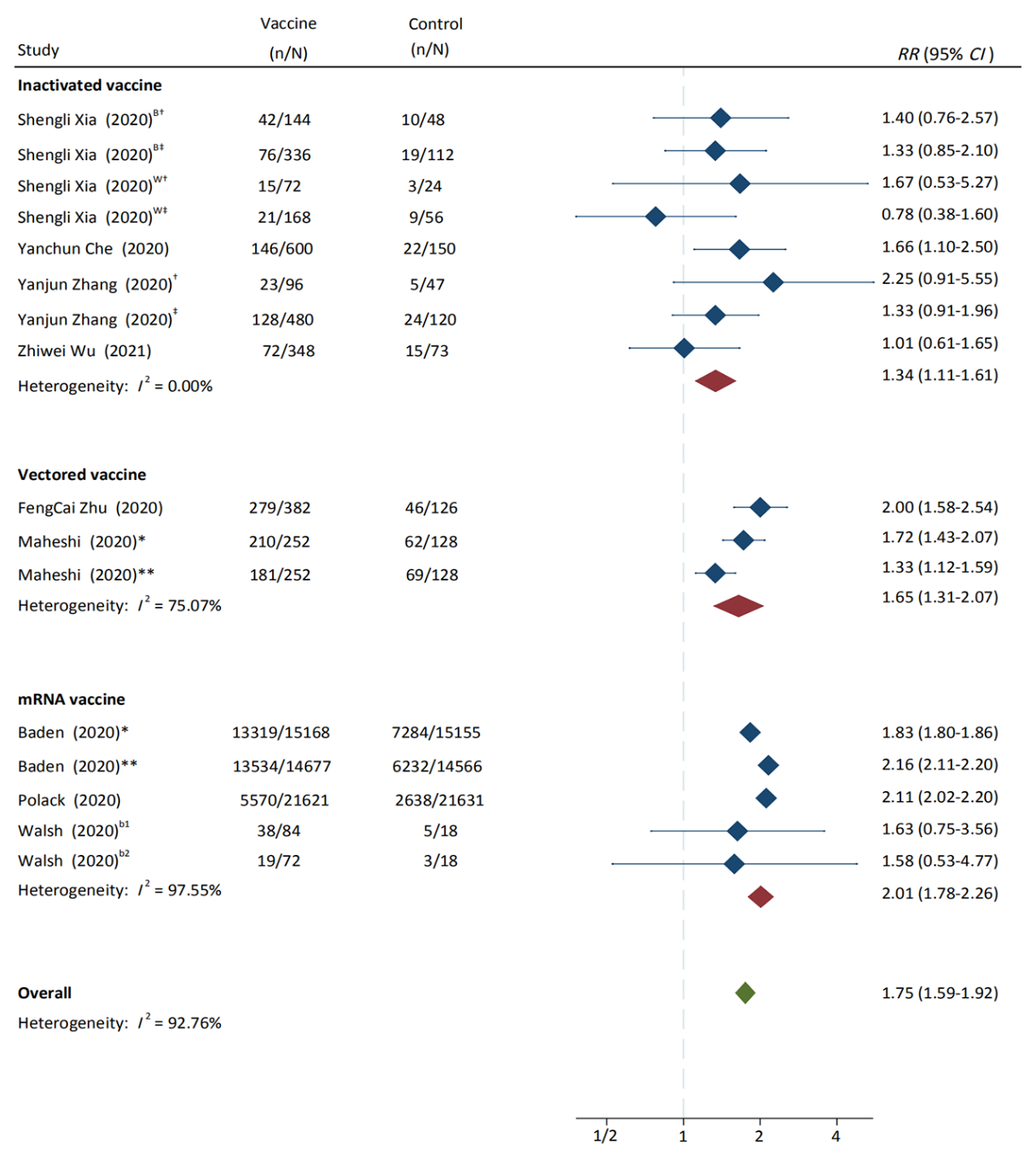


Footnote: ^B^BBIBP-CorV designed by the Beijing Institute of Biological Products; ^W^ CoronaVac designed by the Wuhan Institute of Biological Products; †COVID-19 vaccines in the Phase I Trial; ‡COVID-19 vaccines in the Phase II Trial; *COVID-19 vaccines on first vaccination; **COVID-19 vaccines on second vaccination; ^b1^BNT162b1 in the Phase I Trial; ^b2^BNT162b2 in the Phase I Trial.

*RR*: Risk Ratio**;** *CI*: Confidence Interval; mRNA: Messenger Ribonucleic Acid

**Figure S3. Occurrence of systemic adverse reactions between vaccination group and placebo group**


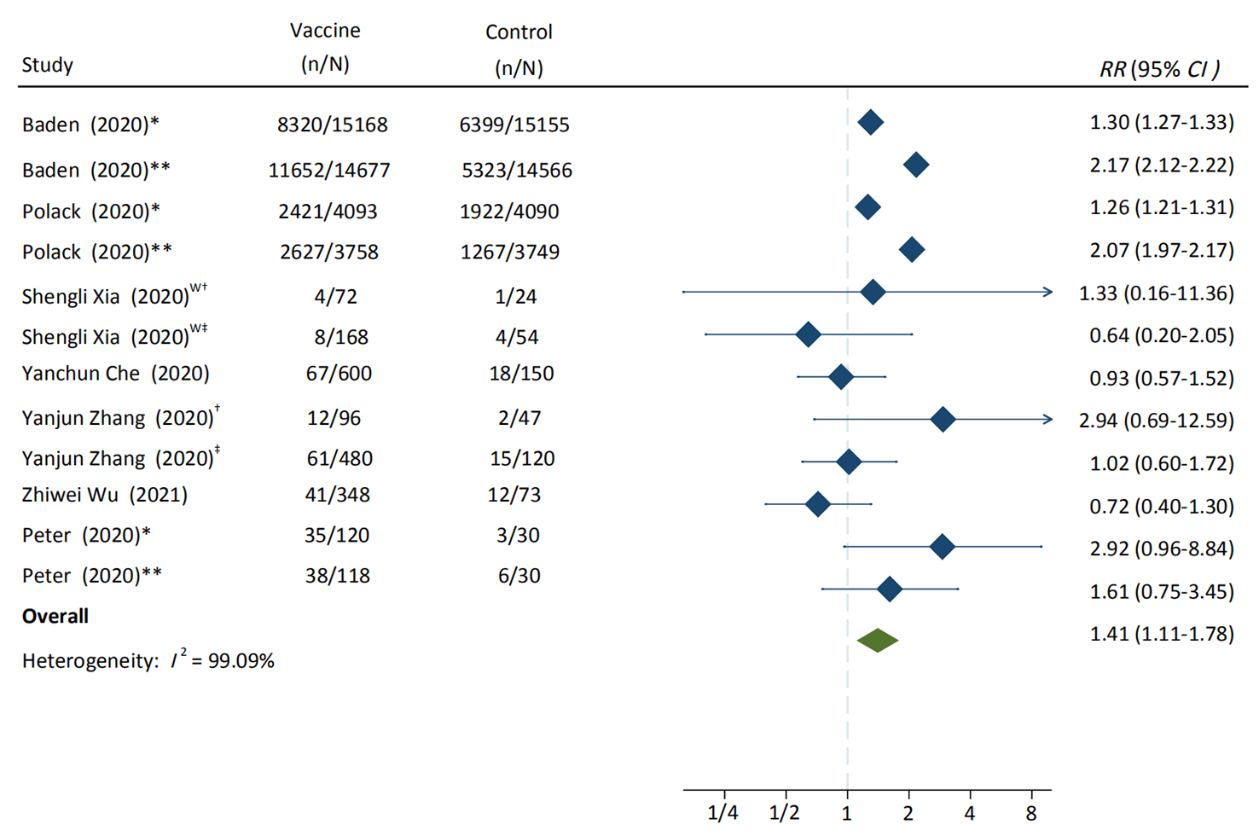


Footnote: ^W^ CoronaVac designed by the Wuhan Institute of Biological Products; †COVID-19 vaccines in the Phase I Trial; ‡COVID-19 vaccines in the Phase II Trial; *COVID-19 vaccines on first vaccination; **COVID-19 vaccines on second vaccination.

*RR*, Risk Ratio**;** *CI*: Confidence Interval.

**Figure S4. Occurrence of local adverse reactions between vaccination group and placebo group**


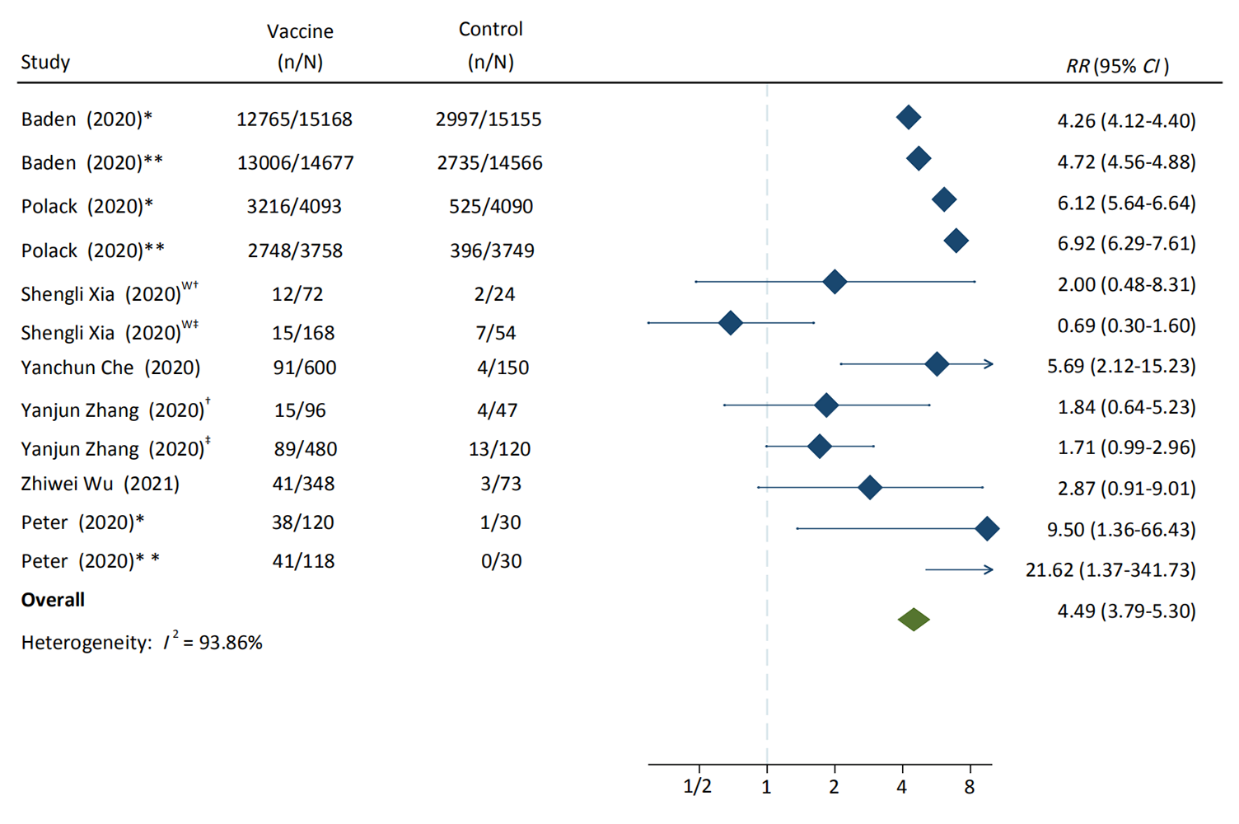


Footnote: ^W^ CoronaVac designed by the Wuhan Institute of Biological Products; †COVID-19 vaccines in the Phase I Trial; ‡COVID-19 vaccines in the Phase II Trial; * COVID-19 vaccines on first vaccination; ** COVID-19 vaccines on second vaccination.

*RR*: Risk Ratio**;** *CI*: Confidence Interval.

**Figure S5. Occurrence of systemic adverse reactions between vaccination group and placebo group subgroup analysis**


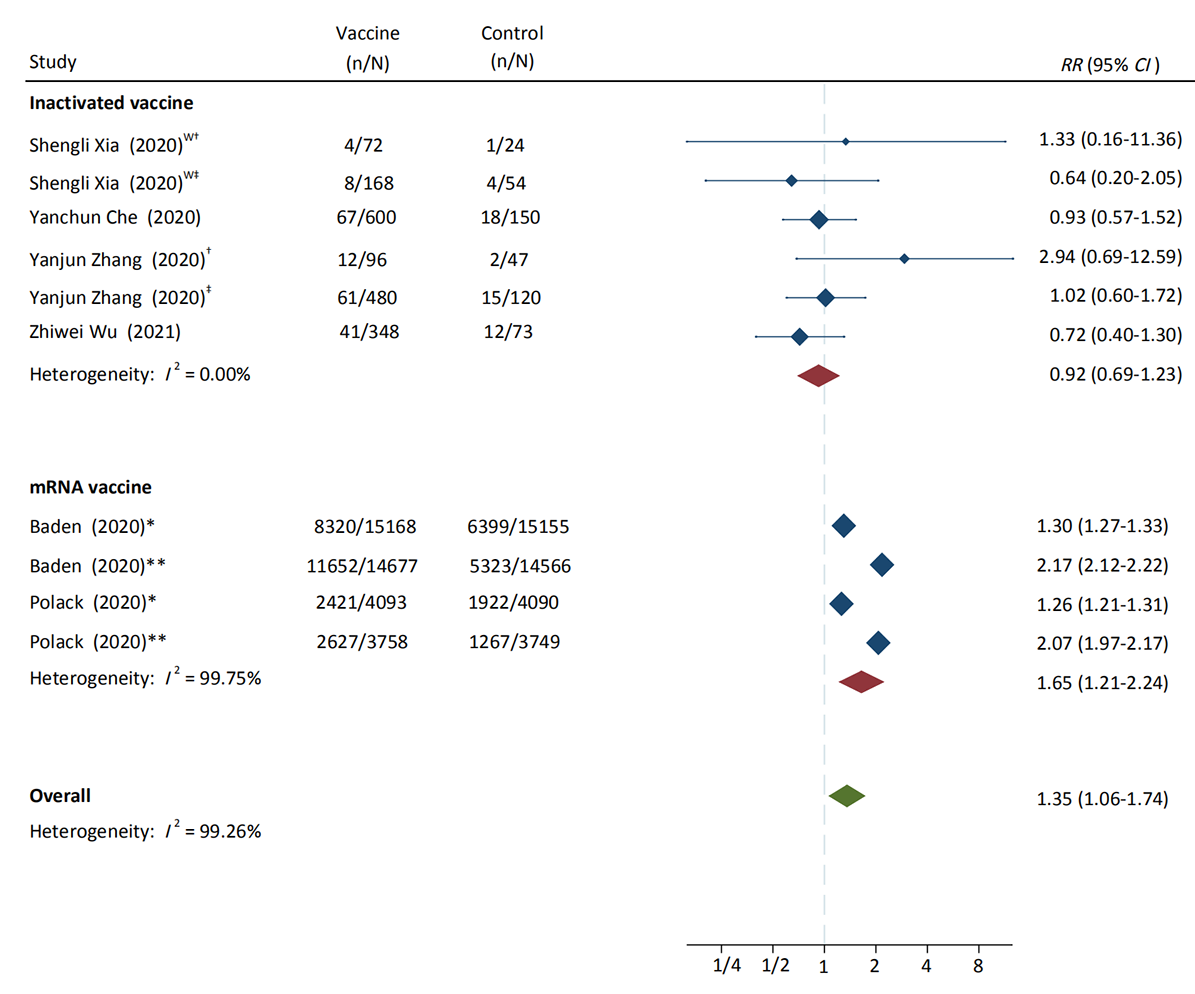


Footnote: ^W^ CoronaVac designed by the Wuhan Institute of Biological Products; †COVID-19 vaccines in the Phase I Trial; ‡COVID-19 vaccines in the Phase II Trial; *COVID-19 vaccines on first vaccination; **COVID-19 vaccines on second vaccination.

*RR*: Risk Ratio**;** *CI*: Confidence Interval; mRNA: Messenger Ribonucleic Acid

**Figure S6. Occurrence of local adverse reactions between vaccination group and placebo group after subgroup analysis**


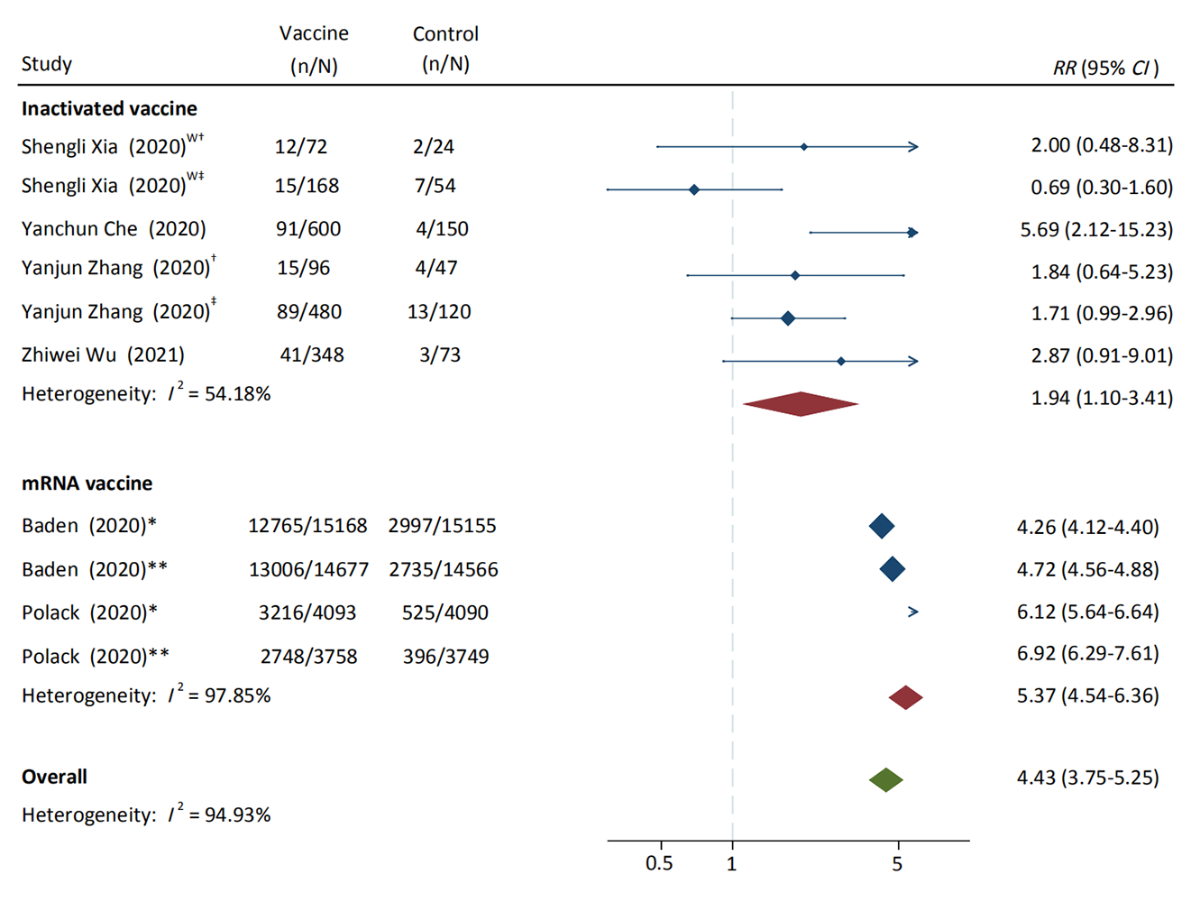


Footnote: ^W^ CoronaVac designed by the Wuhan Institute of Biological Products; †COVID-19 vaccines in the Phase I Trial; ‡COVID-19 vaccines in the Phase II Trial; *COVID-19 vaccines on first vaccination; **COVID-19 vaccines on second vaccination.

*RR*: Risk Ratio**;** *CI*: Confidence Interval; mRNA: Messenger Ribonucleic Acid

**Figure S7. Occurrence of systemic adverse reactions to the inactivated vaccination**


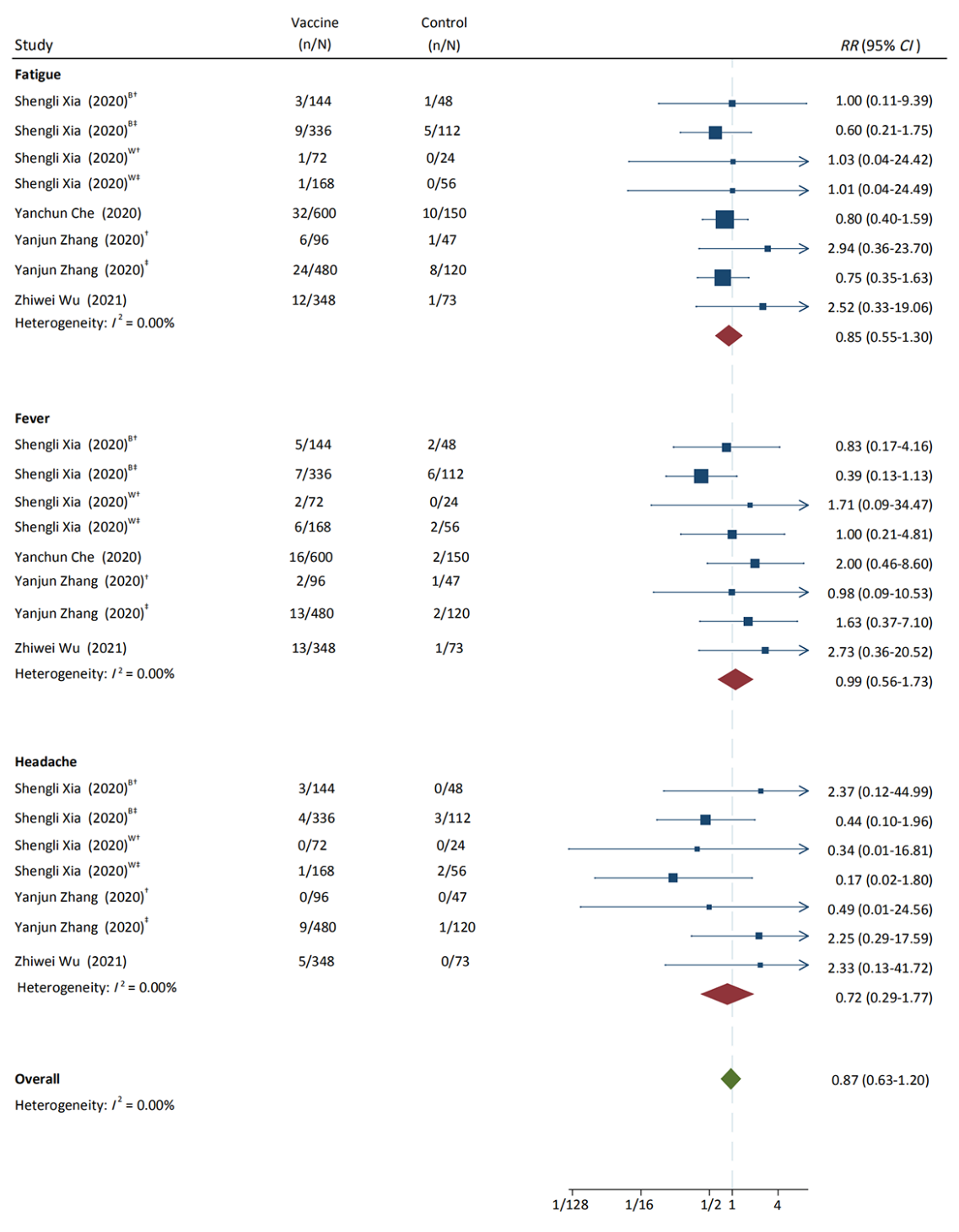


Footnote: ^B^BBIBP-CorV designed by the Beijing Institute of Biological Products; ^W^CoronaVac designed by the Wuhan Institute of Biological Products; †COVID-19 vaccines in the Phase I Trial; ‡COVID-19 vaccines in the Phase II Trial.

*RR*: Risk Ratio**;** *CI*: Confidence Interval.

**Figure S8. Occurrence of local adverse reactions to the inactivated vaccination**

**
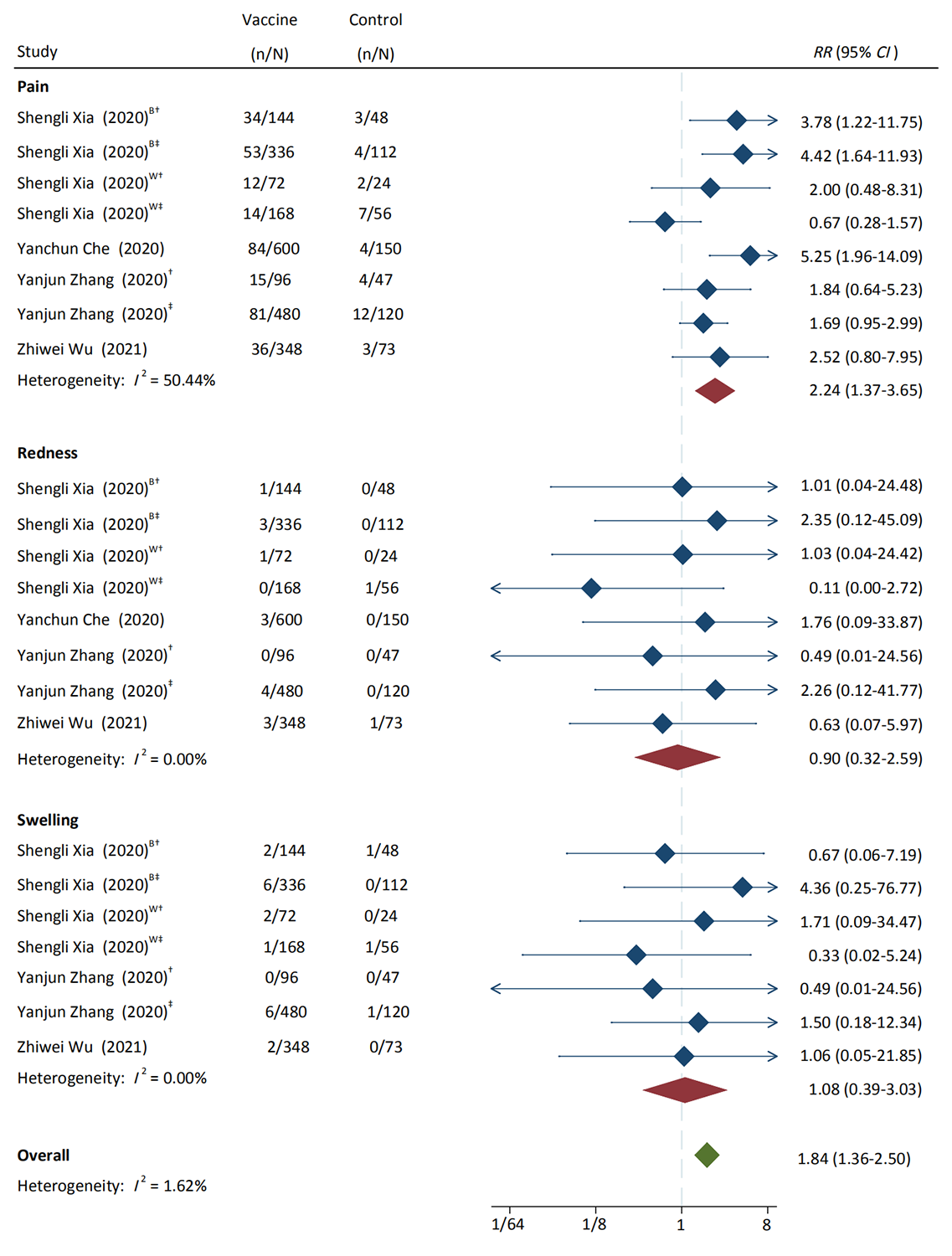
**

Footnote: ^B^BBIBP-CorV designed by the Beijing Institute of Biological Products; ^W^ CoronaVac designed by the Wuhan Institute of Biological Products; †COVID-19 vaccines in the Phase I Trial; ‡COVID-19 vaccines in the Phase II Trial.

*RR*: Risk Ratio**;** *CI*: Confidence Interval.

**Figure S9. Occurrence of systemic adverse reactions to mRNA vaccination**


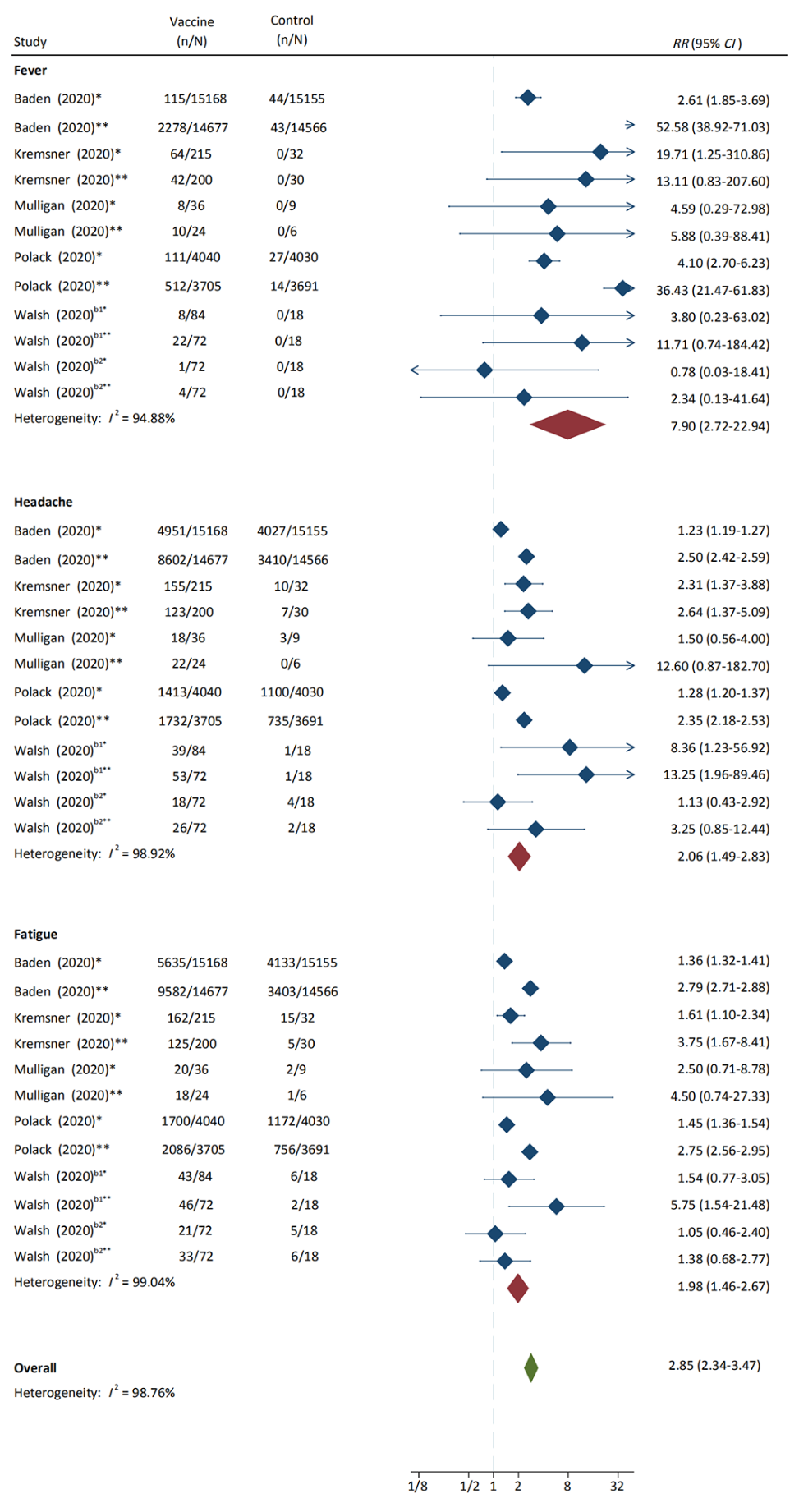


Footnote: * COVID-19 vaccines on first vaccination; **COVID-19 vaccines on second vaccination; ^b1^BNT162b1 in the Phase I Trial; ^b2^BNT162b2 in the Phase II Trial.

*RR*: Risk Ratio**;** *CI*: Confidence Interval.

**Figure S10. Occurrence of local adverse reactions to mRNA vaccination**


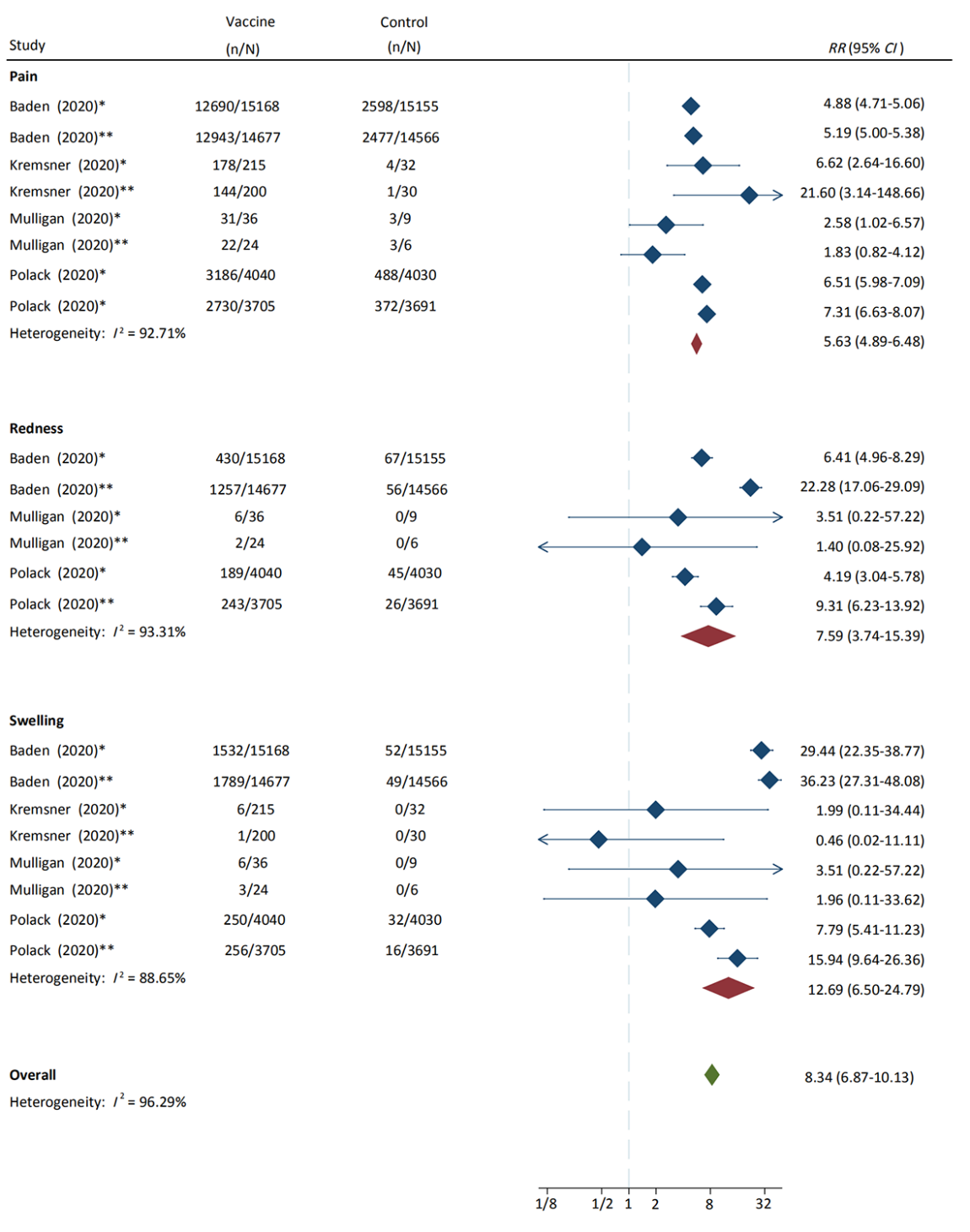


Footnote: * COVID-19 vaccines on first vaccination; **COVID-19 vaccines on second vaccination.

*RR*: Risk Ratio**;** *CI*: Confidence Interval.

**Figure S11. Occurrence of systemic adverse reactions to viral-vector vaccination**


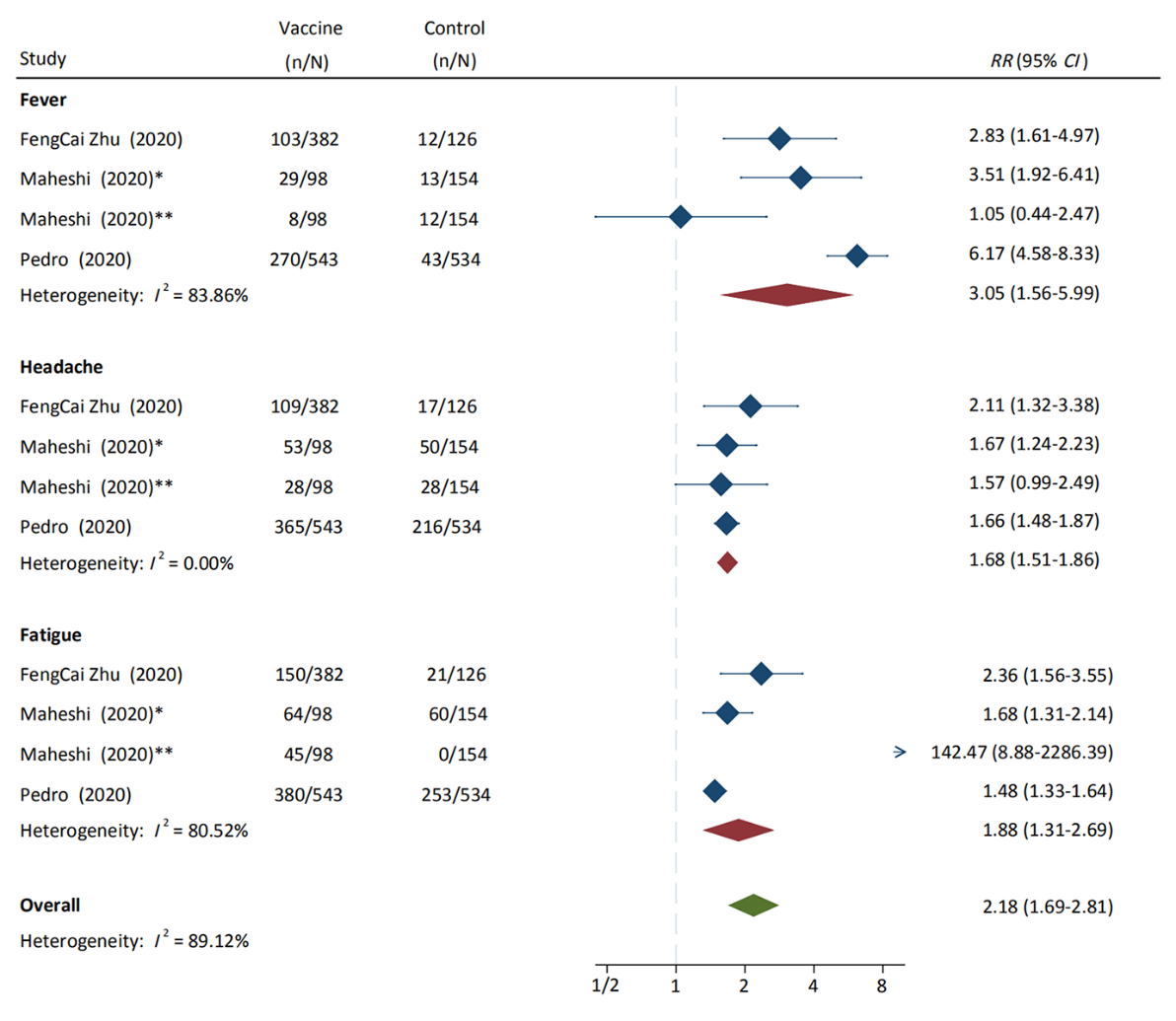


Footnote: * COVID-19 vaccines on first vaccination; **COVID-19 vaccines on second vaccination.

*RR*, Risk Ratio**;** *CI*: Confidence Interval.

**Figure S12. Occurrence of local adverse reactions to viral-vector vaccination**


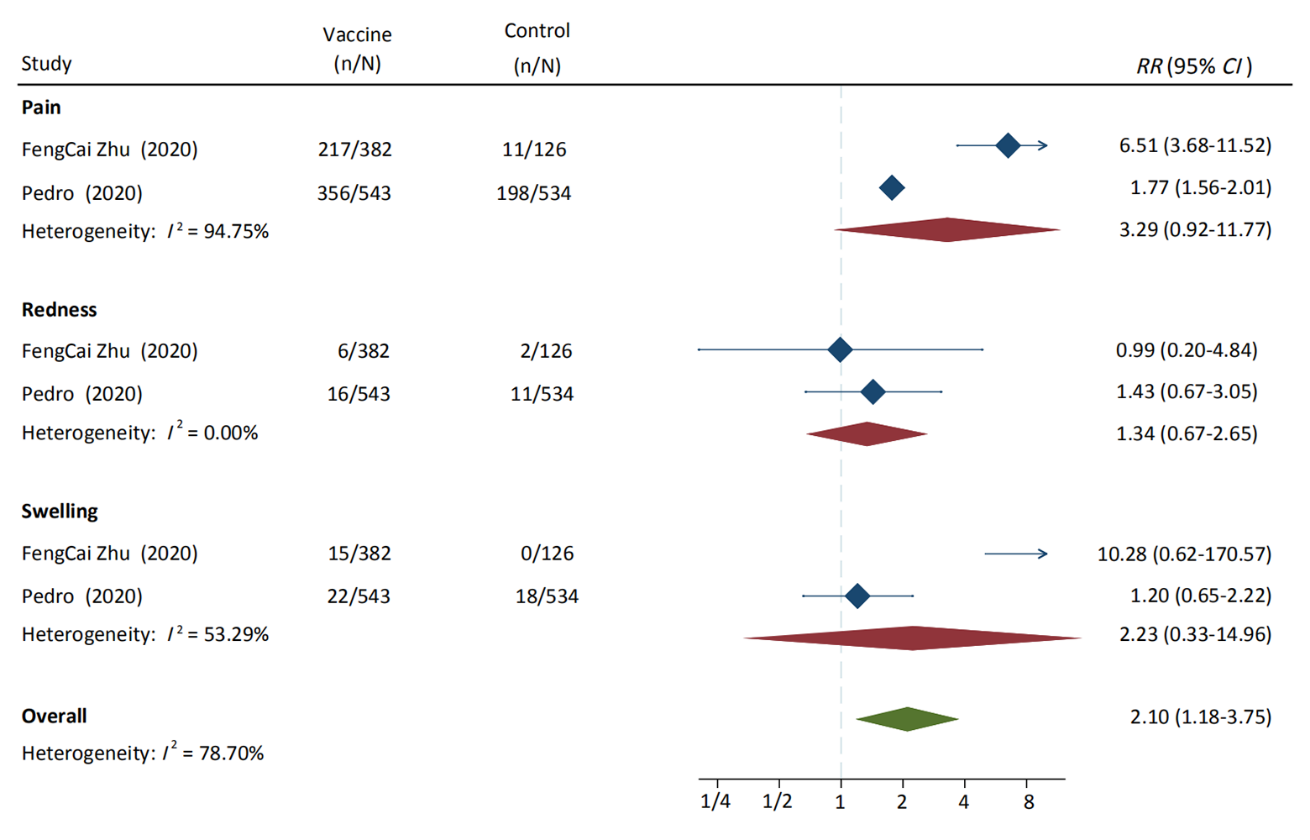


Footnote: *RR*: Risk Ratio**;** *CI*: Confidence Interval.

**Figure S13. Occurrence of adverse reactions to different sample size**


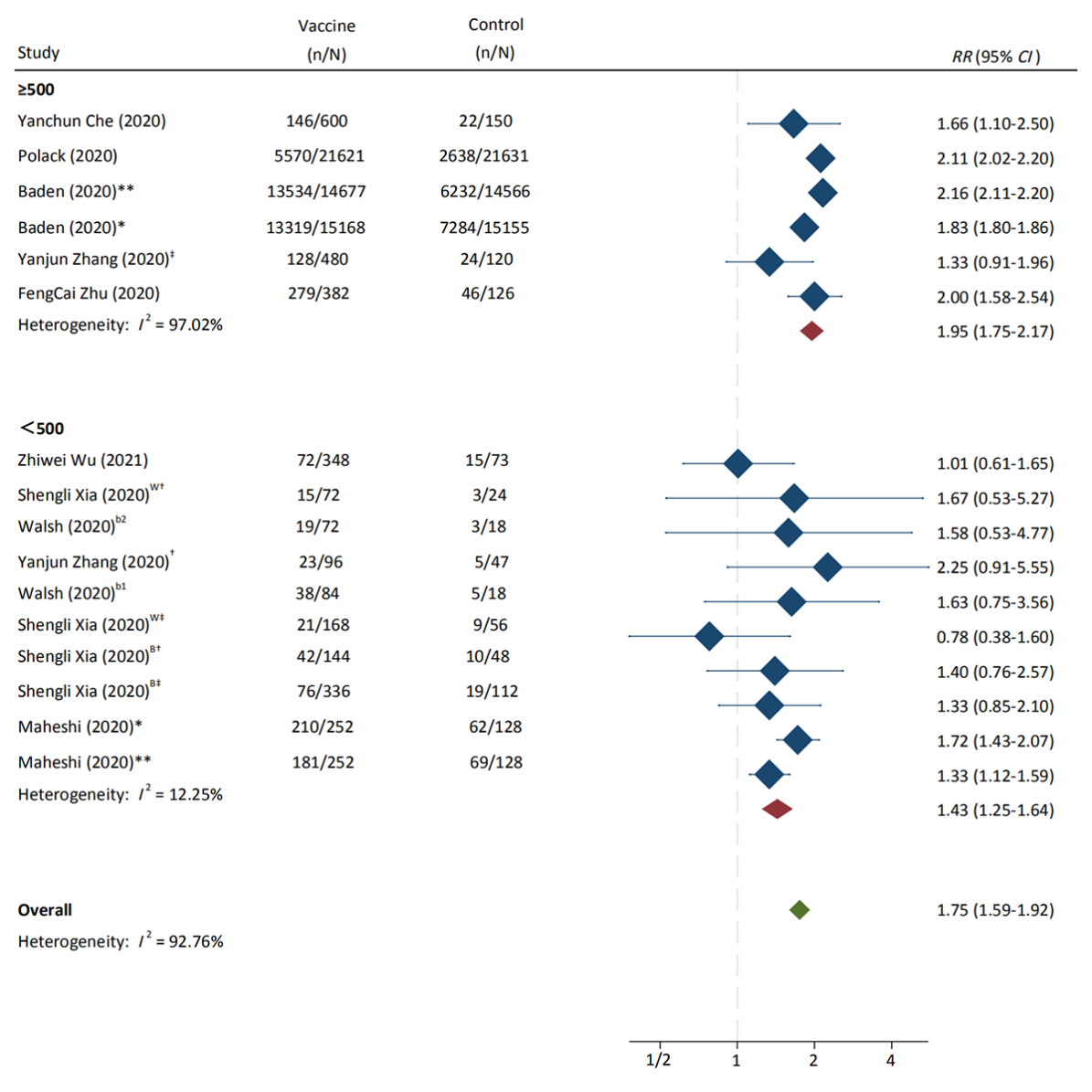


Footnote: ^B^BBIBP-CorV designed by the Beijing Institute of Biological Products; ^W^ CoronaVac designed by the Wuhan Institute of Biological Products; †COVID-19 vaccines in the Phase I Trial; ‡COVID-19 vaccines in the Phase II Trial; *COVID-19 vaccines on first vaccination; **COVID-19 vaccines on second vaccination; ^b1^BNT162b1 in the Phase I Trial; ^b2^BNT162b2 in the Phase I Trial.

*RR*: Risk Ratio**;** *CI*: Confidence Interval.

**Figure S14.** **Occurrence of adverse reactions to different trial phase.**

**
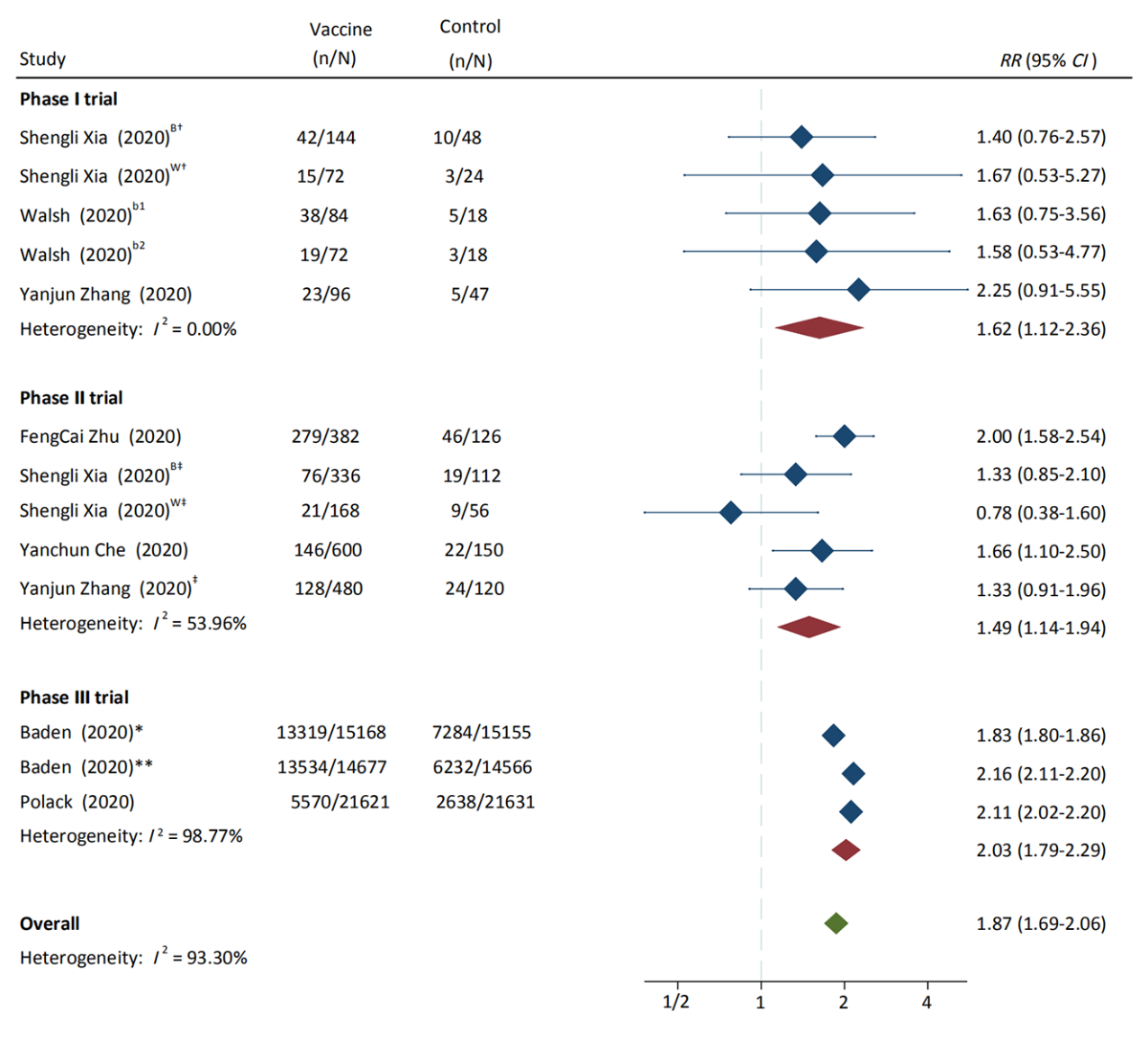
**

Footnote: ^B^BBIBP-CorV designed by the Beijing Institute of Biological Products; ^W^ CoronaVac designed by the Wuhan Institute of Biological Products; †COVID-19 vaccines in the Phase I Trial; ‡COVID-19 vaccines in the Phase II Trial; *COVID-19 vaccines on first vaccination; **COVID-19 vaccines on second vaccination; ^b1^BNT162b1 in the Phase I Trial; ^b2^BNT162b2 in the Phase I Trial.

*RR*: Risk Ratio**;** *CI*: Confidence Interval.

**Figure S15**. **Occurrence of systemic reactions between people aged ≤55 years and aged ≥56 years**


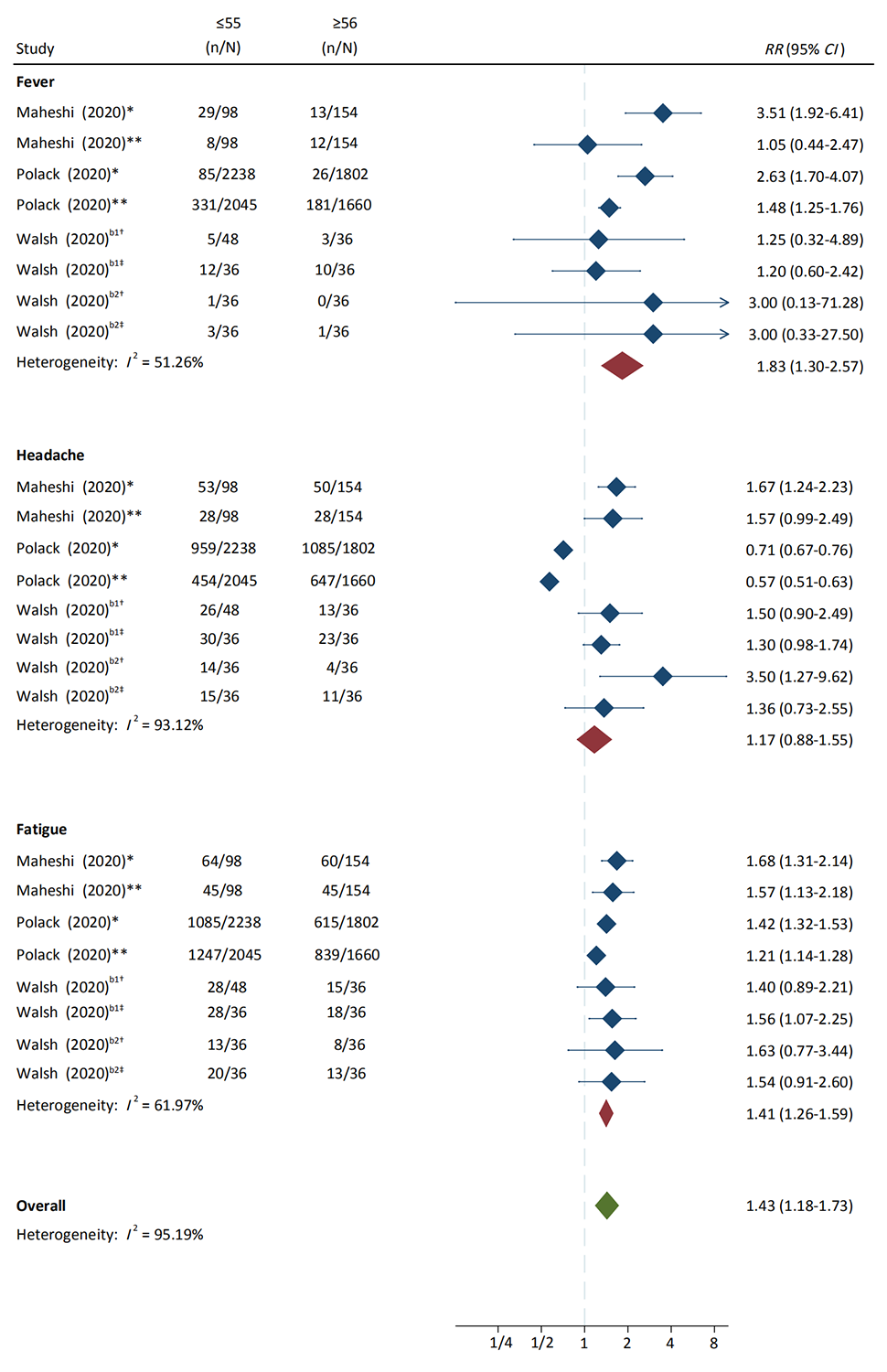


Footnote: †COVID-19 vaccines in the Phase I Trial; ‡COVID-19 vaccines in the Phase II Trial; * COVID-19 vaccines on first vaccination; **COVID-19 vaccines on second vaccination; ^b1^BNT162b1 in the Phase I Trial; ^b2^BNT162b2 in the Phase II Trial.

*RR*: Risk Ratio**;** *CI*: Confidence Interval.

**Figure S16**. **Occurrence of local reactions between people aged ≤55 years and aged ≥56 years**


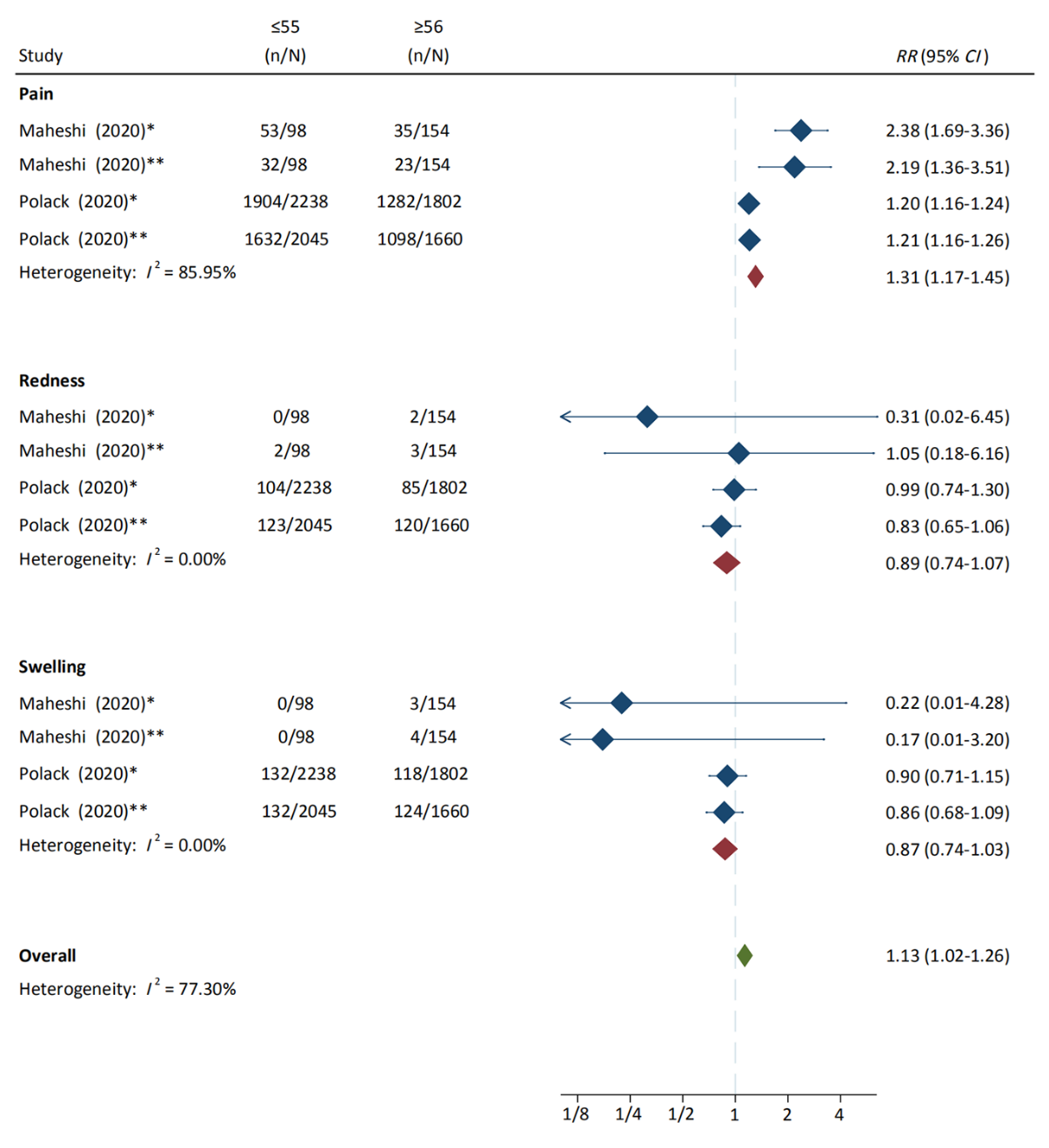


Footnote: * COVID-19 vaccines on first vaccination; ** COVID-19 vaccines on second vaccination.

*RR*: Risk Ratio**;** *CI*: Confidence Interval.

**Figure S17. Funnel plot of the risk ratio estimates for the total adverse reactions vs the standard errors.**

**
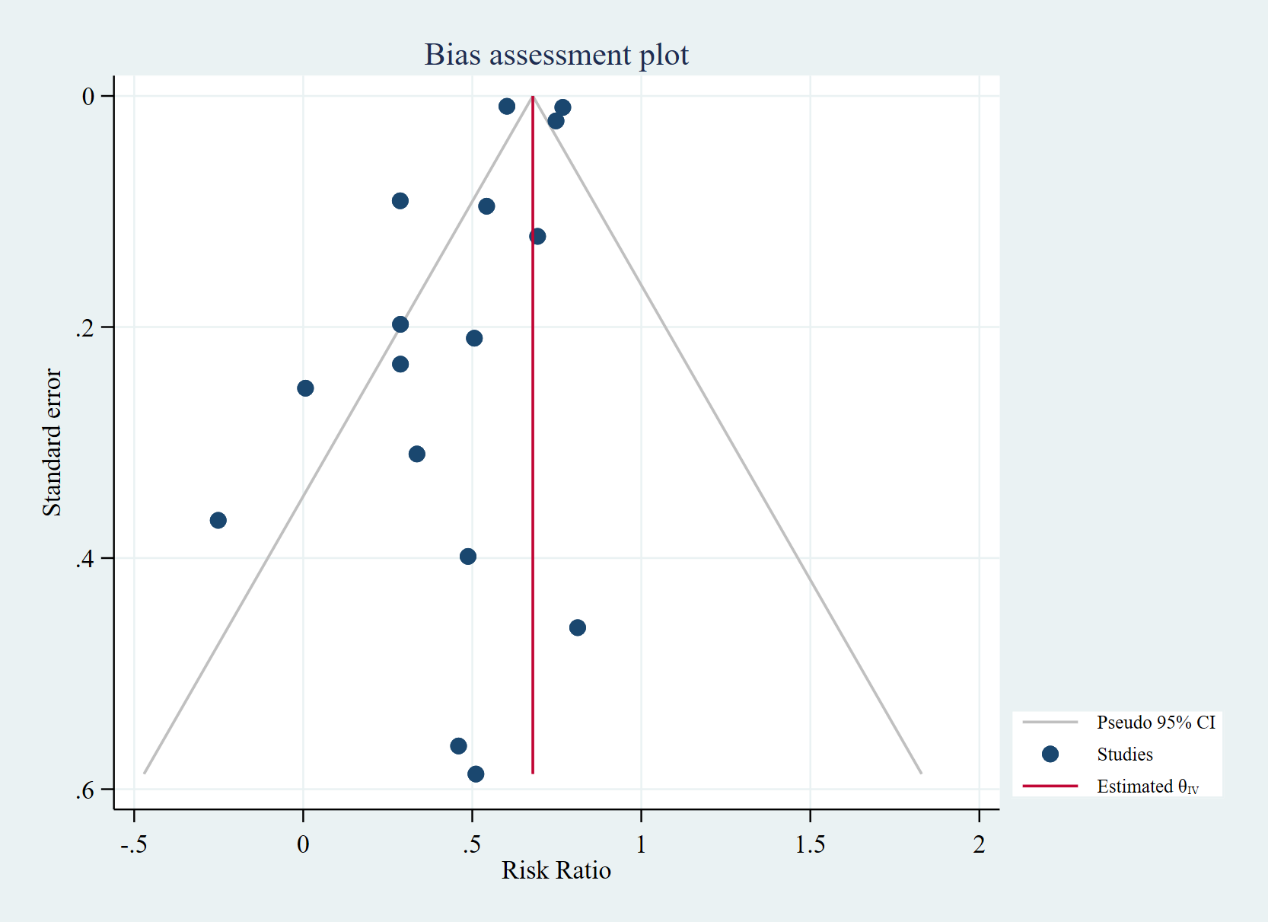
**

Footnote: Egger test for publication bias =-1.130, *P* = 0.0054
